# Characterization of radiations‐induced genomic structural variations in *Arabidopsis thaliana*

**DOI:** 10.1111/tpj.17180

**Published:** 2024-12-01

**Authors:** Salimata Ousmane Sall, Abdelmalek Alioua, Sébastien Staerck, Stéfanie Graindorge, Michel Pellicioli, Jacky Schuler, Catherine Galindo, Quentin Raffy, Marc Rousseau, Jean Molinier

**Affiliations:** ^1^ Institut de biologie moléculaire des plantes du CNRS 12 rue du Général Zimmer 67000 Strasbourg France; ^2^ Institut pluridisciplinaire Hubert Curien Campus de Cronenbourg 23 rue Loess BP 28 67037 Strasbourg Cedex France; ^3^ Present address: Ecole Nationale Supérieure d'Ingénieurs de Caen Laboratoire de physique corpusculaire 6 Boulevard du maréchal Juin 14050 Caen Cedex 4 France

**Keywords:** genotoxic stress, ionizing radiations, non‐ionizing radiations, structural variants, genome stability, genome flexibility, DSB repair

## Abstract

DNA, is assaulted by endogenous and exogenous agents that lead to the formation of damage. In order to maintain genome integrity DNA repair pathways must be efficiently activated to prevent mutations and deleterious chromosomal rearrangements. Conversely, genome rearrangement is also necessary to allow genetic diversity and evolution. The antagonist interaction between maintenance of genome integrity and rearrangements determines genome shape and organization. Therefore, it is of great interest to understand how the whole linear genome structure behaves upon formation and repair of DNA damage. For this, we used long reads sequencing technology to identify and to characterize genomic structural variations (SV) of wild‐type *Arabidopsis thaliana* somatic cells exposed either to UV‐B, to UV‐C or to protons irradiations. We found that genomic regions located in heterochromatin are more prone to form SVs than those located in euchromatin, highlighting that genome stability differs along the chromosome. This holds true in Arabidopsis plants deficient for the expression of master regulators of the DNA damage response (DDR), ATM (Ataxia‐telangiectasia‐mutated) and ATR (Ataxia‐telangiectasia‐mutated and Rad3‐related), suggesting that independent and alternative surveillance processes exist to maintain integrity in genic regions. Finally, the analysis of the radiations‐induced deleted regions allowed determining that exposure to UV‐B, UV‐C and protons induced the microhomology‐mediated end joining mechanism (MMEJ) and that both ATM and ATR repress this repair pathway.

## Significance Statement

Exposure to genotoxic stress activates DNA damage response (DDR) and challenges genome integrity. Using third generation sequencing technology, we characterized alterations of the linear genome structure of Arabidopsis plants treated with non‐ionizing (UV‐B, UV‐C) and ionizing radiations (protons). We identified, in wild type and DDR deficient plants, that heterochromatin is more prone to form structural variations (SVs) than euchromatin, highlighting that genome stability and flexibility vary along chromosomes.

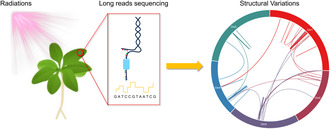

## INTRODUCTION

DNA is the support of the genetic information and defines the genome of living organisms. Eukaryotic nuclear genome is organized through the linear order of genetic entities such as protein coding genes (PCG), intergenic regions (IR), transposable elements (TE) and repeats. Genome size results from the antagonist interactions between processes regulating its stability and allowing rearrangements (defined as genome flexibility; Schubert & Vu, 2016). Indeed, genome stability is necessary to ensure integrity while genome flexibility allows genetic diversity and evolution (Bhadra et al., 2023; Melamed‐Bessudo et al., 2016). Variations in genome size and organization occur through changes in ploidy level (i.e., whole genome duplication) or during stress‐ or development‐induced structural variations (SV; Masuda et al., 2017; Schubert & Vu, 2016; Zhang et al., 2023). All these changes contribute to the reshaping of the genome and occur with different dynamics. Indeed, particular genomic regions display higher structural variability, such as centromeres/pericentromeres, containing large amounts of repeats and TE, respectively (Lian et al., 2024; Naish & Henderson, 2024).

Plants, like most of the living organisms, are exposed to numerous environmental cues that can generate point mutations and alter genome structure. The release of TE silencing can lead to their mobilization and to transposition events affecting genome organization and structure (Roquis et al., 2021; Zhang et al., 2023). Endogenous agents (i.e., secondary metabolites) and exposure to environmental cues (i.e., high light) induce the formation of DNA damage (Mehta & Haber, 2014; Molinier, 2017; Yousefzadeh et al., 2021). In order to maintain genome integrity and to prevent deleterious chromosomal rearrangements, several different processes are activated. Transcriptional and post‐transcriptional gene silencing, restrict TE mobilization, and DNA repair pathways allow maintenance of genome integrity from single nucleotide to dozens of base pairs (Bergis‐Ser et al., 2024; Herbst et al., 2024). The DNA damage response (DDR) is orchestrated by 2 phosphatidyl‐inositol 3‐kinase‐like (PI3) protein kinases: ATR (Ataxia‐telangiectasia‐mutated and Rad3‐related) and ATM (Ataxia‐telangiectasia‐mutated; Nisa et al., 2019). ATM is the main DNA Double Strand Break (DSB) signal transducer (Bergis‐Ser et al., 2024; Herbst et al., 2024; Szurman‐Zubrzycka et al., 2023) whereas ATR triggers DDR during replication stress, upon exposure to UV and to DNA modifying agents (Szurman‐Zubrzycka et al., 2023). ATM and ATR phosphorylate specific factors, albeit they also share some common targets (Roitinger et al., 2015; Shiloh, 2001). ATM and ATR deficient Arabidopsis plants exhibit hypersensitivity to DSB‐ and replicative stress‐inducing agents, respectively (Culligan et al., 2006; Garcia et al., 2000, 2003). ATM mutant plants are sterile (Garcia et al., 2003). However, the combination of both mutations allows the development of viable plants but does not restore the fertility (Vespa et al., 2005).

DSBs are induced upon exposure to genotoxic agents (i.e., non‐ionizing and ionizing radiations) and to biotic/abiotic stresses (Kovalchuk et al., 2003; Lucht et al., 2002; Mehta & Haber, 2014; Molinier et al., 2005). DSBs are also formed by repair intermediates of different types of damage (Reitz et al., 2023; Sobol et al., 2003), during replication (Schuermann et al., 2009; Waterworth et al., 2011), during transpositions events (Hedges & Deininger, 2007) and during meiosis by the specific endonuclease SPO11 (Grelon et al., 2001). DSB is a deleterious DNA damage that must be efficiently repaired. DSBs are processed by 2 main pathways: Non‐Homologous End‐Joining (NHEJ) and Homologous Recombination (HR; Puchta, 2005; Schuermann et al., 2005). NHEJ is an error prone process that ligates break ends with most of the time a loss of genetic information (Puchta, 2005). Conversely, HR is an accurate repair mechanism that uses a homologous sequence found on the sister chromatid or within the homologous chromosome (Puchta, 2005). The predominant DSB repair pathway used in plants is NHEJ (Puchta, 2005). Other DSB repair pathways rely on homology‐directed repair or DNA synthesis (Puchta, 2005; Schubert & Vu, 2016). The Microhomology‐mediated end joining (MMEJ), involves alignment of micro‐homologous sequences (2–20 bp) in the vicinity of the DNA break and leads to variable sizes of deletions (Puchta, 2005; Schubert & Vu, 2016). The synthesis‐dependent strand‐annealing (SDSA) pathway uses homologous DNA templates by strand displacement (Puchta, 2005; Schubert & Vu, 2016). SDSA allows mostly error free repair although insertions originating from various sequences templates could occur (Puchta, 2005).

In somatic plant cells, several different approaches have been developed to monitor the use of these different DSB repair pathways. Exogenous templates (i.e., linearized plasmids; Dubest et al., 2002; Orel & Puchta, 2003; Puchta & Hohn, 1991), transgenes (Molinier et al., 2004; Swoboda et al., 1994), CRISPR Cas9, combined with high throughput short reads sequencing, revealed how NHEJ/HR are used and led to the identification of various chromosomal rearrangements (Samach et al., 2023; Vu et al., 2017). Indeed, these strategies allowed the characterization of the DSB repair pathway choice (Vu et al., 2017) and of the factors involved, directly or indirectly, in the different repair processes (Vu et al., 2017). We highly gained in knowledge with the use of CRISPR Cas9 to induce DNA breaks within particular genomic/epigenomic contexts in order to characterize the outcome of repair (Vu et al., 2017). Nevertheless, it remains to be deciphered how the whole linear genome organization behaves upon exposure to different types of DNA damaging agents. Indeed, DNA damage formation and the choice of the DNA repair may vary according to the genomic and epigenomic contexts (i.e., chromatin compaction level; Johann to Berens & Molinier, 2020) and thus could influence the balance between genome stability and flexibility (Johann to Berens & Molinier, 2020). The genome wide analysis of structural variations (SV) would provide an overview of the chromosomal rearrangements that may have occurred in each genomic/epigenomic contexts independently of a targeted sequence. This is made possible with the development of the third‐generation sequencing methods producing long reads (>50 kb; Marx, 2023) and allowing an accurate coverage of the genome, including repetitive sequences (Naish et al., 2021, Naish & Henderson, 2024). This paves the way for the genome wide identification and characterization of SV.

In this study, we addressed the question of the induction of genomic SV in somatic Arabidopsis plant cells, upon exposure to ionizing (accelerated protons) and non‐ionizing radiations (UV‐B and UV‐C). Using long reads sequencing technology, we first characterized, in three independent biological replicates, the putative SV of WT Arabidopsis plants originating from our collection of seeds in comparison with the publicly available reference genome TAIR10 (https://www.arabidopsis.org/). This allowed defining the pedigree of our plants to further characterize, qualitatively and quantitatively, the repertoire of SV induced in WT Arabidopsis plants exposed to UV‐B, UV‐C and protons. We identified that ionizing and non‐ionizing radiations triggered to same types of chromosomal rearrangements and that constitutive heterochromatin is more prone to form these SV than other part of the (epi)genome.

We also analyzed the linear genome structure of Arabidopsis plants deficient for the expression of ATM and ATR, the master regulators of the DDR. We found that euchromatic genic regions are less prone to be rearranged than heterochromatic TE‐IR, highlighting that different and complex genome surveillance mechanisms exist along the genome, even in the absence of the main DDR factors.

Finally, the long reads sequencing data allowed determining to which extents, NHEJ and homology‐directed DSB repair have been used. We characterized radiation‐induced MMEJ events and identified that ATM and ATR repress this repair pathway.

This study provides the genome‐wide profiles of rearrangements induced upon exposure to non‐ionizing and ionizing radiations, and documents that genome stability and flexibility differ between centromeres/pericentromeres and chromosome arms.

## RESULTS

### Experimental design

**Figure 1:**
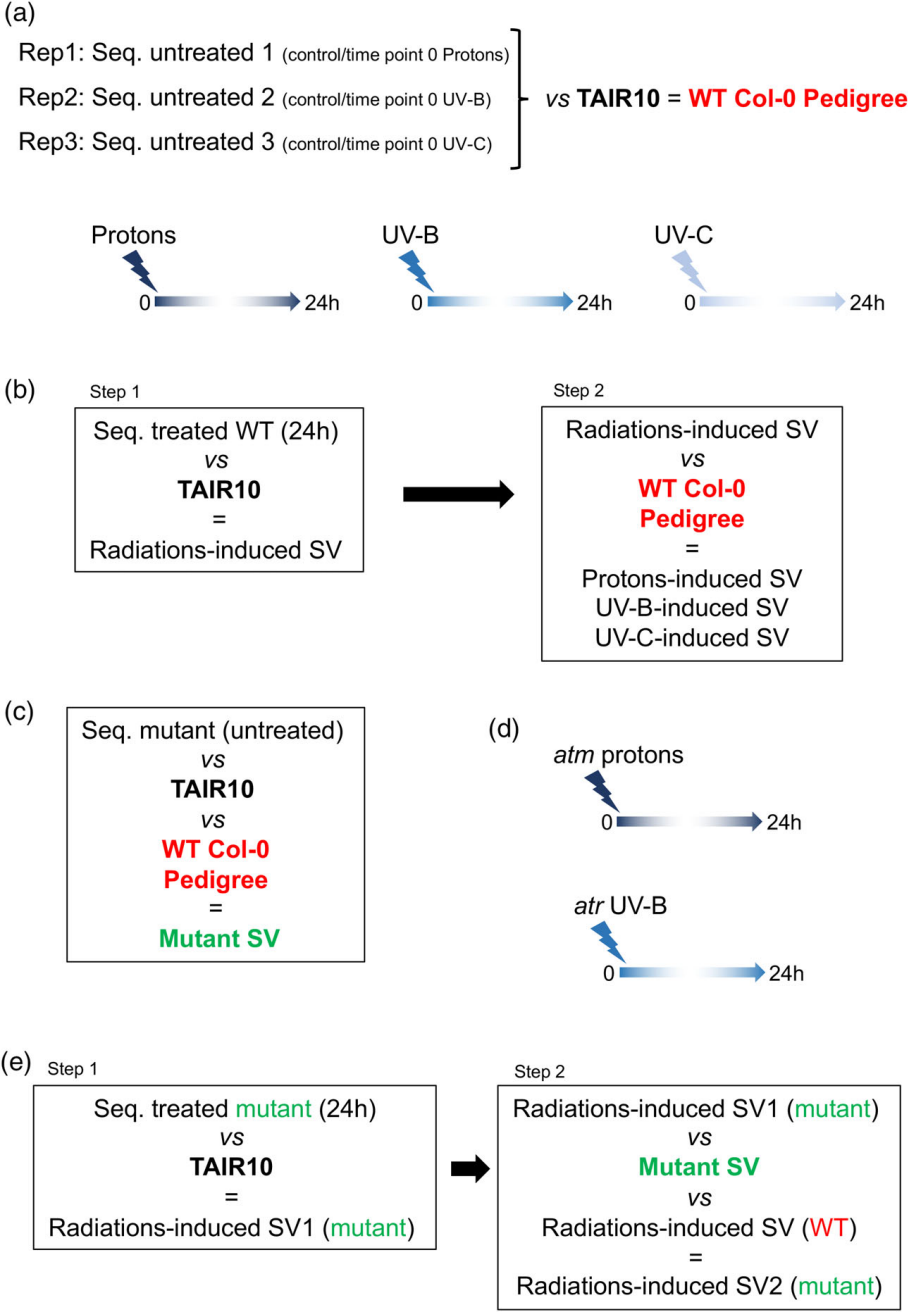
Experimental design. (a) WT, Col‐0, Arabidopsis plants were irradiated with either protons, UV‐B or UV‐C. Then, 24 h upon each treatment, plant material was harvested to determine radiations‐induced SV using long reads sequencing. Each untreated control (time point 0) of the 3 different types of irradiations, have been used to characterize the pedigree of our collection of WT (Col‐0) Arabidopsis seeds. The comparison of the genomic sequences (seq.) with the TAIR10 reference genome allows defining the SVs of the plants originating from our collection of WT (Col‐0) Arabidopsis seeds. The total number of SV defines our pedigree and is thus used as reference for further experiments. (b) Schematic representation of the approach designed to characterize UV‐B‐, UV‐C‐ and protons‐induced SV in WT plants. Step 1: long reads sequences (seq.) of treated plants are compared to the TAIR10 reference genome to identify radiations‐induced SV. Step 2: SVs of our pedigree are subtracted to radiations‐induced SV to determine the UV‐B‐, UV‐C‐ and protons‐induced SVs. (c) Schematic representation of the approach designed to characterize SV in DDR deficient plants. Long reads sequences (seq.) of untreated *atm* and *atr* plants are compared to the TAIR10 reference genome to identify SV in mutant plants. (d) *atm* and *atr*, plants were irradiated with protons and UV‐B, respectively. Then, 24 h upon each treatment, plant material was harvested to determine radiations‐induced SV using long reads sequencing. (e) Same as (b) for *atr* UV‐B‐ and *atm* protons‐treated plants.

In order to characterize genotoxic stress‐induced SV in different Arabidopsis genotypes, we needed to define to which genomic data we had to refer to. First, we characterized whether WT Arabidopsis plants originating from our collection of seeds contained genomic SV such as insertions, deletions, duplications, inversions and inversions duplications, relative to the TAIR10 reference genome (https://www.arabidopsis.org/; Figure 1a). For this, we performed long reads sequencing of the genomic DNA prepared from 3 independent biological replicates of untreated WT (Col‐0) Arabidopsis plants. Importantly, these biological replicates correspond to the untreated control of each genotoxic treatment (Figure 1a). This allowed defining our plant pedigree (Figure 1a). Second, we identified the radiations‐induced SVs by sequences comparison with the TAIR10 reference genome (Figure 1b: step1) and upon subtraction of the total SVs found in our pedigree (Figure 1b: step2). This allowed the identification of SV for each source of radiations (Figure 1b). Third, we determined SV in DDR mutant plants using long reads sequencing data obtained from *atm*, *atr* and *atm atr* untreated plants compared with the TAIR10 reference genome and with our pedigree. This allowed determining *atm*, *atr* and *atm atr* SVs (Figure 1c). Finally, we identified the radiations‐induced SV in both *atm*‐ and *atr*‐treated plants relative to the TAIR10 reference genome and upon subtraction with the SV found in the corresponding untreated mutant and in WT treated plants (Figure 1d,e). This experimental design is thought to take into account SV originating from all genetic backgrounds (WT and DDR deficient plants) in order to identify SV induced by the different sources of radiations.

### Characterization of WT plants pedigree

**Figure 2:**
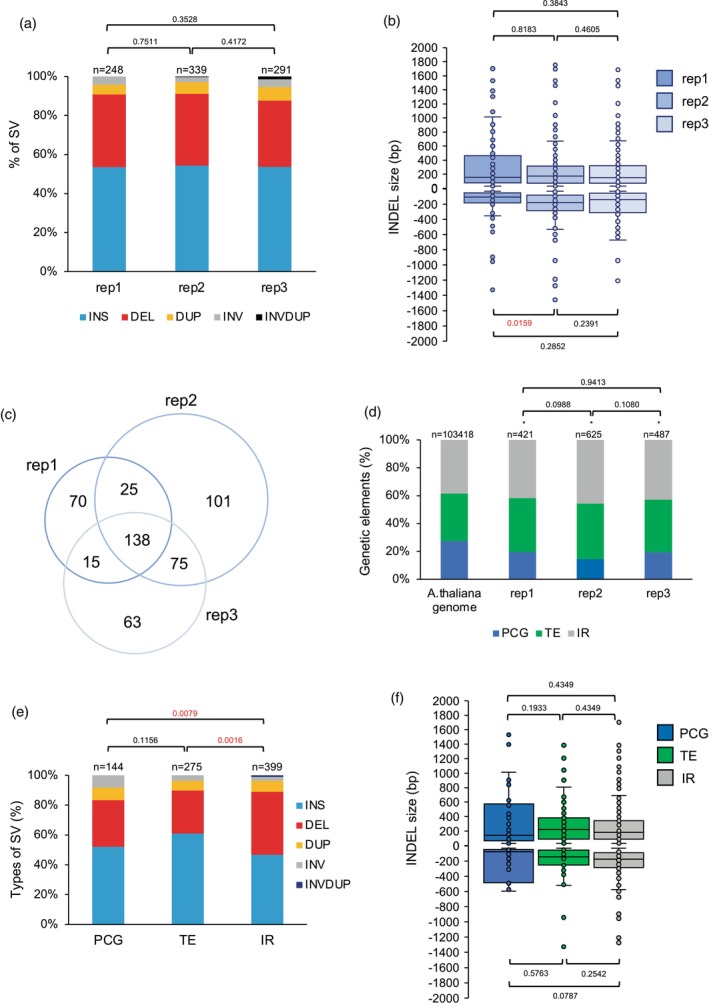
Characterization of the structural variations in WT Arabidopsis plants. (a) Histogram representing the distribution of the different types of genomic SV identified in each of the three independent biological replicates of WT (Col‐0) Arabidopsis plants. DEL, deletion; DUP, duplication; INS, insertion; INV, inversion; INVDUP, inversion duplication. *n* = total number of SV. Exact *P* values are shown (Chi‐squared test). (b) Box plots representing the size of the INDELs identified in each of the three independent biological replicates. Exact *P* values are shown (Mann Whitney Wilcoxon test). (c) Venn diagram representing the overlap of SV between the 3 independent biological replicates. (d) Histogram representing the distribution of the genetic elements (IR, intergenic regions; PCG, protein coding genes; TE, transposable elements) exhibiting SV in each of the three independent biological replicates. The distribution of the genetic elements in the *Arabidopsis thaliana* genome is shown. Exact *P* values are shown, *P < 0.01 versus *A. thaliana* genome (Chi‐squared test). *n* = total number of genetic elements containing SV. (e) Histogram showing the distribution of the genomic SV (DEL, deletion; DUP, duplication; INS, insertion; INV, inversion; INVDUP, inversion duplication) identified in the genetic elements of the 3 biological replicates (IR, intergenic regions; PCG, protein coding genes; TE, transposable elements). Exact *P* values are shown (Chi‐squared test). (f) Box plots representing the INDELs sizes identified in the genetic elements (IR, intergenic regions; PCG, protein coding genes; TE, transposable elements). In boxplots, the central line and bounds of the box represent the median and the 25th and 75th quartiles, respectively. The whiskers represent 1.5× interquartile range of the lower or upper quartiles. Exact *P* values are shown (Mann Whitney Wilcoxon test).

In order to characterize SVs that may contain the somatic cells of WT (Col‐0) untreated plants, we compared the long reads sequencing data obtained from three independent biological replicates with the TAIR10 reference genome. In each replicate we identified 248, 339 and 291 SVs, such as insertions, deletions, duplications, inversions and inversions‐duplications (Figure 2a). The distribution of the different types of SV does not vary between replicates (Figure 2a). INDELS (insertions–deletions) are predominantly represented (>80%; Figure 2a) and their sizes do not significantly differ between samples (Figure 2b) suggesting a limited variability among samples.

The comparison of the SV found in each replicate shows that 138 are common between the 3 samples, 7–22% are shared between 2 replicates, and 21–29% are unique to each replicate (Figure 2c). This shows that a core of SV exists in the Arabidopsis plants originating from our collection of seeds but also that some SV are present at lower frequencies.

**Figure 3:**
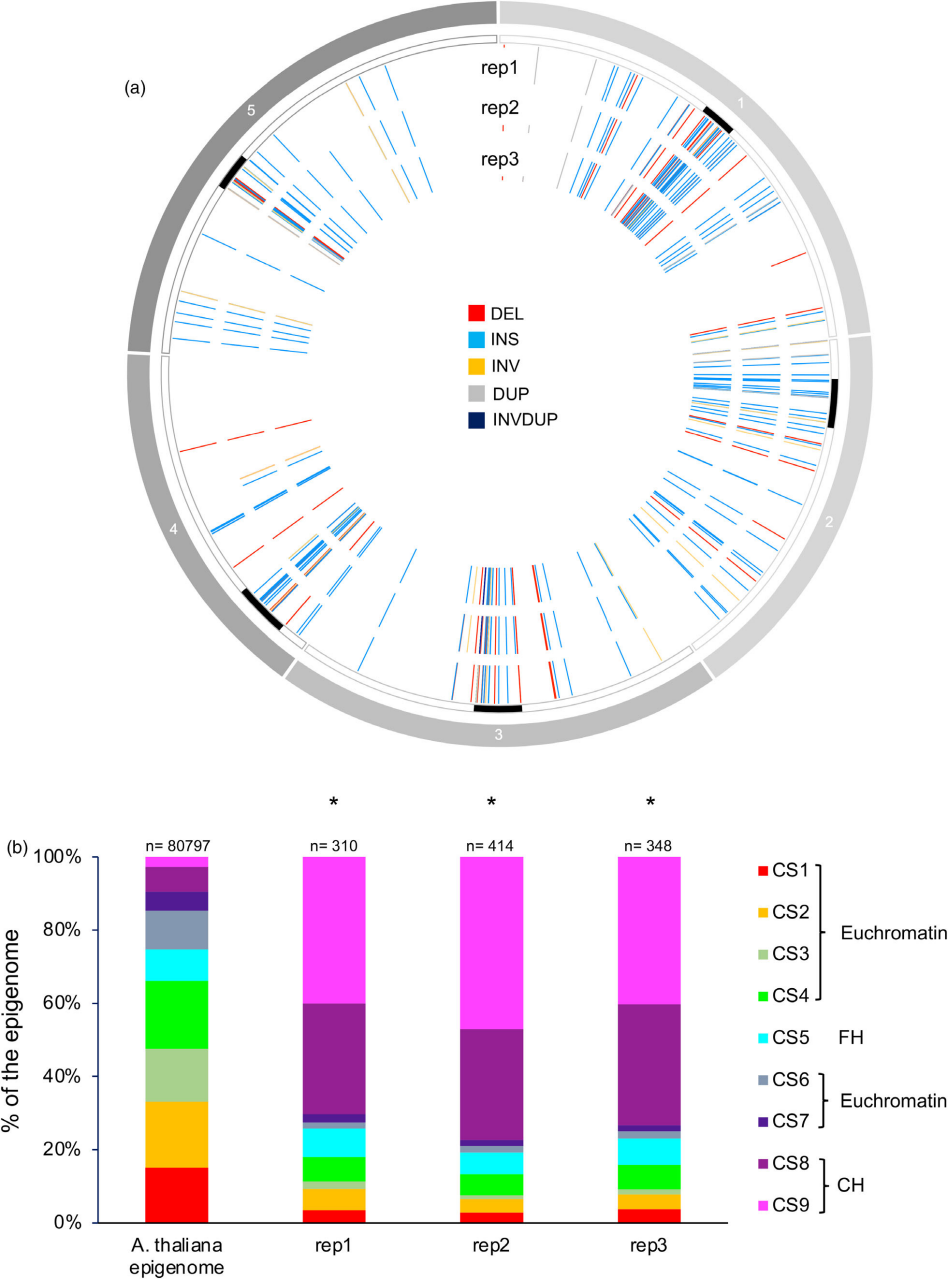
Genomic location and epigenomic features of the structural variations identified in WT Arabidopsis plants. (a) Circos representation of genomic SV (DEL, deletion; DUP, duplication; INS, insertion; INV, inversion; INVDUP, inversion duplication) identified in each independent biological replicate. Black rectangles represent the centromeres. (b) Histogram representing the distribution of the chromatin states (CS) overlapping with the SV identified in each independent biological replicates. Chi‐squared test *P < 0.01 compared to the CS distribution in the Arabidopsis epigenome (Sequeira‐Mendes et al., 2014). CH, constitutive heterochromatin; FH, facultative heterochromatin. *n* = total number of CS containing SV.

The distribution of the genetic elements: Protein coding genes (PCG); transposable elements (TE) and intergenic regions (IR), affected by SVs, is not significantly different between replicates (Figure 2d). Importantly, TE and IR represent more than 80% of the location of the SV (Figure 2d), stressing that these genetic entities are more prone to be rearranged than PCG. Deletions occurred more often in IR than in TE and PCG, but their sizes remain similar between genetic entities (Figure 2e,f). The overrepresentation of TE and IR, exhibiting SV, suggests that particular genetic elements might preferentially undergo rearrangements and that particular genomic/epigenomic features might exist. Thus, we compared the location of the identified SV with (i) the coordinates of centromeric regions/chromosomes arms and (ii) the publicly available Arabidopsis epigenomic landscape defined as chromatin states (CS; Sequeira‐Mendes et al., 2014). Most of the SV (>70%) overlaps with centromeric and pericentromeric regions (Figure 3a) and with the repressive chromatin states: CS8 and 9 (Sequeira‐Mendes et al., 2014; Figure 3b). This highlights that constitutive heterochromatin, enriched in repetitive elements, is more prone to form SV than euchromatin. LTR/Gypsy and DNA TE superfamilies are significantly overrepresented among all TE containing SV (Figure S1) indicating that these types of TE exhibit more flexibility than others.

Hence, the use of long reads sequencing allows the identification of genomic SV in Arabidopsis WT plants originating from our collection of seeds in comparison with the Arabidopsis TAIR10 reference genome. These results indicate that genomic variability exists between plants and with the reference TAIR10 genome. Moreover, it shows that these changes occur predominantly in constitutive heterochromatin containing high number of repetitive sequences that are thought to be more prone to be rearranged (Lian et al., 2024; Naish & Henderson, 2024).

Therefore, these genomic data will be used as references to qualitatively and quantitatively determine the effect of genotoxic stress exposure on genome integrity of WT Arabidopsis plants.

### Exposure to radiations induces structural variations, predominantly in constitutive heterochromatin

The use of long reads sequencing of 3 independent biological replicates of WT Arabidopsis plants originating from our collection of seeds allowed determining their pedigree. We used this as reference, to quantitatively and qualitatively characterize genotoxic stress‐induced SVs. We treated WT Arabidopsis plants with non‐lethal doses of either non‐ionizing (UV‐B, UV‐C) or ionizing radiations (protons). UV‐B and UV‐C induce photoproducts (Molinier, 2017), DSB (Molinier et al., 2004; Peak & Peak, 1990; Ries et al., 2000) and to a lower extent oxidatively induced DNA damage (UV‐B; Cadet et al., 2015). Exposure to ionizing radiations, such as protons, mostly leads to the formation of reactive oxygen species (ROS) and to DSB (Kim et al., 2019; Ward, 1988). Importantly, the use of these sources of radiations allows an acute exposure of plants to the genotoxic agent that facilitates the choice of the time point to determine the outcome of repair. It differs from treatments with chemicals (i.e., cisplatin) for which the kinetics of uptake and the time window of the formation of damage are much more difficult to define.

Long reads sequencing was performed on genomic DNA prepared from samples harvested 24 h upon irradiation, when DNA is thought to be repaired (Figure 1a). In a first step, the radiations‐induced SV have been characterized according to the TAIR 10 reference genome (Figure 1b: step 1). In a second step, to retrieve protons‐, UV‐B‐ and UV‐C‐induced SV, according to our pedigree, the total amount of SV identified in the 3 untreated replicates (248 + 339 + 291 = 878) have been subtracted (Figure 1b: step 2).

**Figure 4:**
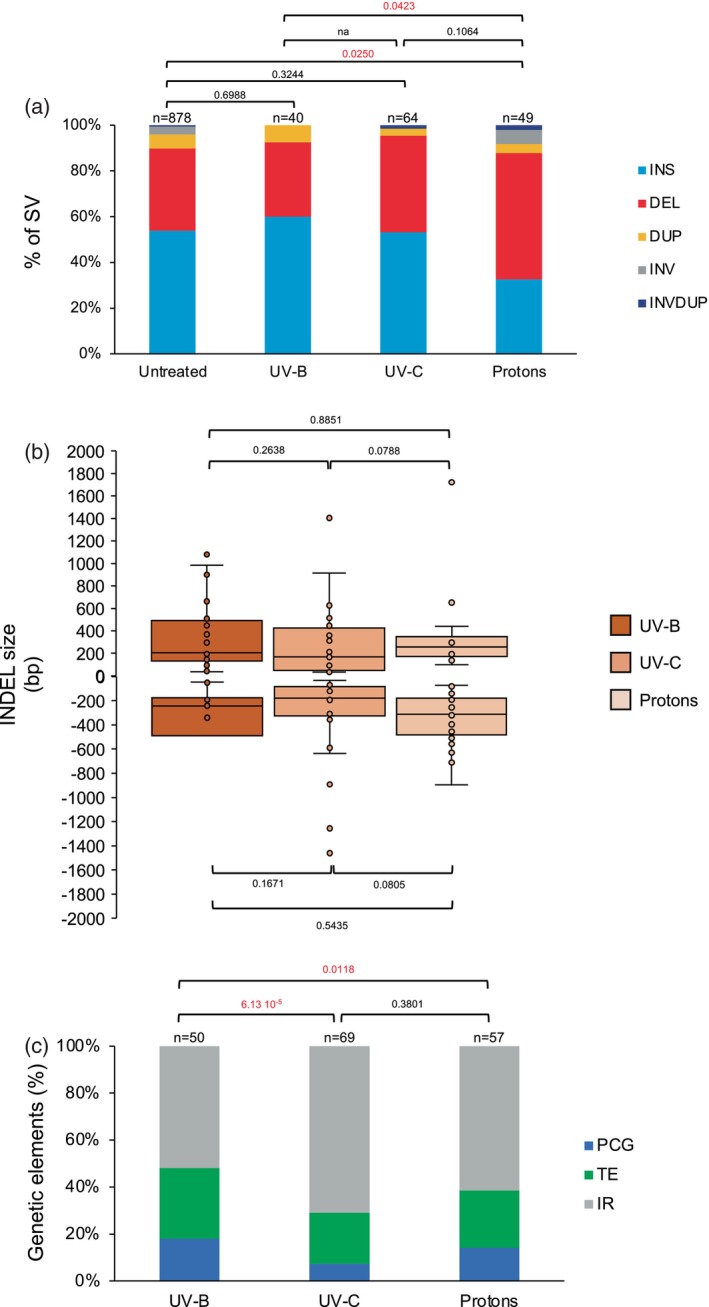
Characterization of the radiation‐induced genomic structural variations in WT Arabidopsis plants. (a) Histogram representing the distribution of the genomic SV identified in untreated WT Arabidopsis plants (relative to the TAIR 10 reference genome) and in plants treated with either UV‐B, UV‐C or protons. DEL, deletion; DUP, duplication; INS, insertion; INV, inversion; INVDUP, inversion duplication. *n* = total number of SV. Exact *P* values are shown (Chi‐squared test). na, non‐applicable. (b) Box plots representing the INDELs sizes identified in WT Arabidopsis plants treated with either UV‐B, UV‐C or protons. In boxplots, the central line and bounds of the box represent the median and the 25th and 75th quartiles, respectively. The whiskers represent 1.5× interquartile range of the lower or upper quartiles. Exact *P* values are shown (Mann‐Whitney Wilcoxon test). (c) Histogram representing the distribution of the genetic elements (IR, intergenic regions; PCG, protein coding genes; TE, transposable elements) exhibiting SV upon exposure to either UV‐B, UV‐C, or protons. Exact *P* values are shown (Chi‐squared test). *n* = total number of genetic elements containing SV.

Exposure of WT Arabidopsis plants to UV‐B, UV‐C or protons led to the formation of 40, 64 and 49 SVs, respectively, and are enriched in INDELS (Figure 4a). Protons treatment induced more deletions than UV‐B or UV‐C treatments (Figure 4a), in relationship with the high energy delivered by ionizing radiations and their deleterious effect on DNA (Cadet et al., 2015; Ward, 1988). Insertions represent more than 50% of the SV induced upon exposure to UV‐B and to UV‐C (Figure 4a). This observation highlights that the outcome of repair differs upon exposure to non‐ionizing and ionizing radiations, likely due to the types of DNA damage induced, and to the repair processes used (Kim et al., 2019; Molinier, 2017). Nevertheless, the sizes of the radiations‐induced INDELs do not differ significantly among treatments (Figure 4b). Both TE and IR are mainly affected by SV (Figure 4c). LTR/Copia (Class I) are predominantly altered upon protons treatment whilst Class II TE (i.e., MuDR) represents the main superfamily exhibiting SV upon non‐ionizing radiation exposure (Figure S2). Thus, the source of irradiation might influence the type of TE superfamily in which SV occurred, likely in relationship with their transcriptional activity and/or their intrinsic features.

INDEL sizes, in the different genetic entities, remain invariable between treatments (Figure S3a). UV‐B treatment leads to a significant higher proportion of SV in PCG (mainly insertions) compared to UV‐C and protons irradiations (Figure 4c; Figure S3b), suggesting that this genotoxic agent preferentially induces variability in certain genetic entities.

Given that insertions represent a good proportion of the SV (Figure 4a), their detailed analyses would provide information about the origin of the inserted sequences. Indeed, transposition can lead to de novo insertions in the genome (Muñoz‐López & García‐Pérez, 2010). In addition, different types of sequences (within the same chromosome or between chromosomes) can be used as template for synthesis‐dependent repair (i.e., SDSA) and as filler DNA for repair (Gorbunova & Levy, 1997). We found that most of the inserted sequences originated from TE and IR (Figure S4a), highlighting that these genetic entities are more prone to be used as template for insertions. None of the analyzed insertions revealed a *per se* transposition event, but only truncated TE. In protons‐irradiated plants we found more insertions originating from PCG (Figure S4a), showing that ionizing radiation would rather trigger the use of PCG regions as template for insertions.

We also determined which genetic elements have inserted into PCG, TE and IR. Although the numbers are quite low (between 3 and 20 events), we found a trend to have insertions originating from PCG into PCG, from TE into TE/IR and from IR into IR/TE, with a preference for an intrachromosomal origin (Figures S4b–d and S5). These results highlight that genomic regions, in the vicinity of the DNA breaks, are likely used a template. This could be either due to the linear 1D organization or 3D genome structure.

**Figure 5:**
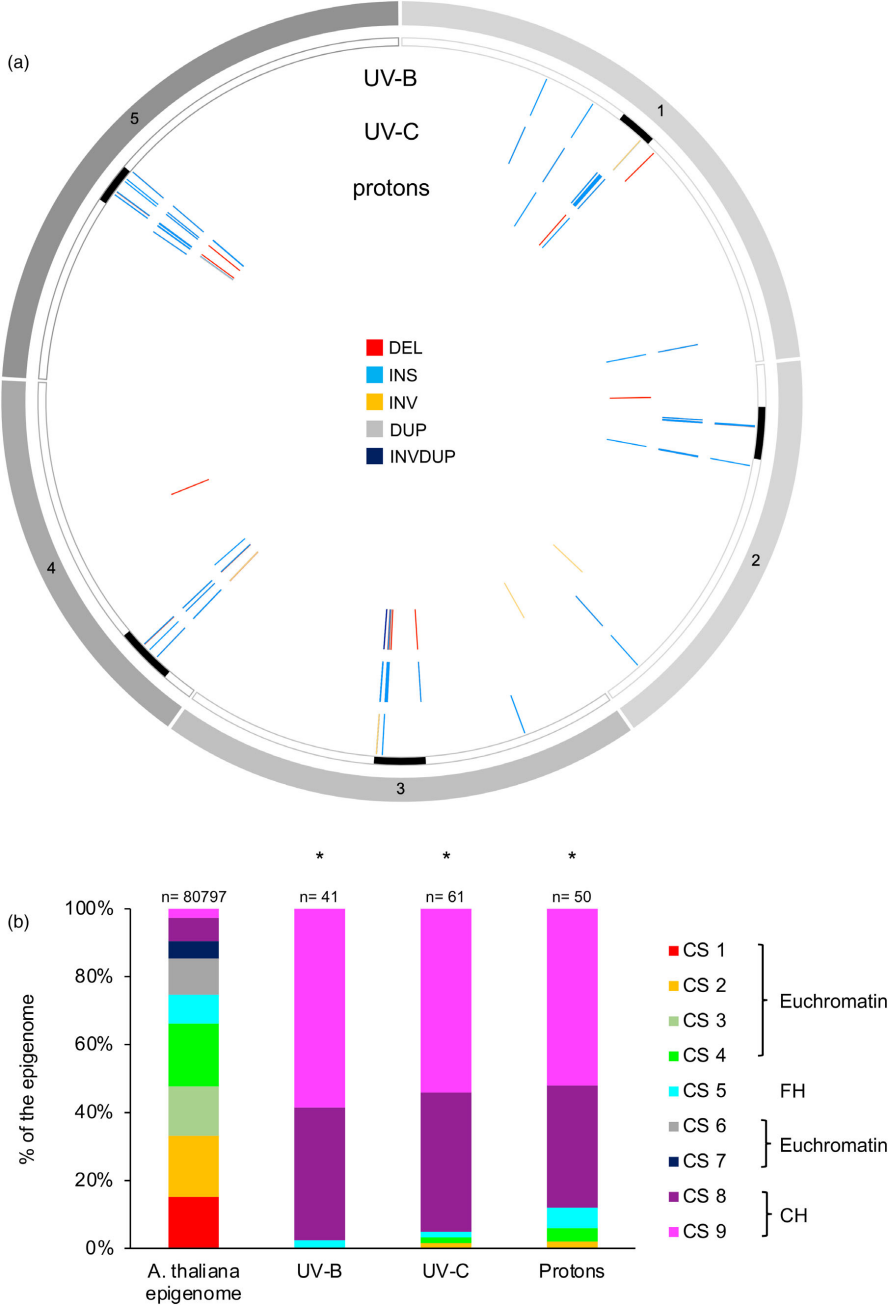
Genomic locations and epigenomic features of genomic structural variations identified in WT Arabidopsis plants irradiated with either UV‐B, UV‐C or protons. (a) Circos representation of genomic SV (DEL, deletion; DUP, duplication; INS, insertion; INV, inversion; INVDUP, inversion duplication) identified upon exposure of WT Arabidopsis plants to either UV‐B, UV‐C or protons. Black rectangles represent the centromeres. (b) Histogram representing the distribution of the chromatin states (CS) overlapping with the SV identified upon exposure to either UV‐B, UV‐C or protons. Chi‐squared test *P < 0.01 compared to the CS distribution in the Arabidopsis epigenome (Sequeira‐Mendes et al., 2014). *n* = total number of CS containing SV.

We investigated whether non‐ionizing and ionizing radiations would have triggered the formation of SV in particular chromatin contexts. In all treatments, SV are located predominantly in centromeric‐pericentromeric regions overlapping with CS8 and 9 (Figure 5a,b), indicating that constitutive heterochromatin is more prone to exhibit structural variability than euchromatin.

The Arabidopsis genome contains regions, called hotspots of rearrangements (HOT), that would facilitate evolutionary responses to rapidly changing environmental challenges (Jiao & Schneeberger, 2020). Thus, we determined whether non‐ionizing and ionizing radiations have led to the formation of SV in such genomic regions. We found 103 radiations‐induced SV (>67% of the SV), that overlap with HOT regions (Figure S6a,b), suggesting that these genomic regions exhibiting flexibility upon exposure to environmental cues and/or genotoxic stress, may contain particular features.

Altogether, the long reads whole genome sequencing of plants exposed to different types of radiations uncovered that constitutive heterochromatin is more prone to form SV than other part of the (epi)genome and that particular regions (i.e., HOT regions) exhibit more flexibility than others.

### Control of genome integrity by ATM and ATR

The PI3‐like kinases, ATM and ATR, activate the DDR to maintain genome integrity in the face of endogenous or exogenous exposures to genotoxic agents (Shiloh, 2001). In order to better define the role of these kinases in the maintenance of genome linear structure, we performed the Oxford Nanopore Technology (ONT) sequencing of *atm*, *atr* single mutant plants and of *atm atr* double mutant plants. In single *atr* and *atm* mutant plants, SV have been determined in comparison with the TAIR10 reference genome (Figure 1c). In double *atm atr* mutant plants, SV have been retrieved from the comparison with the TAIR10 reference genome and with each single mutant plants (Figure 1c).

**Figure 6:**
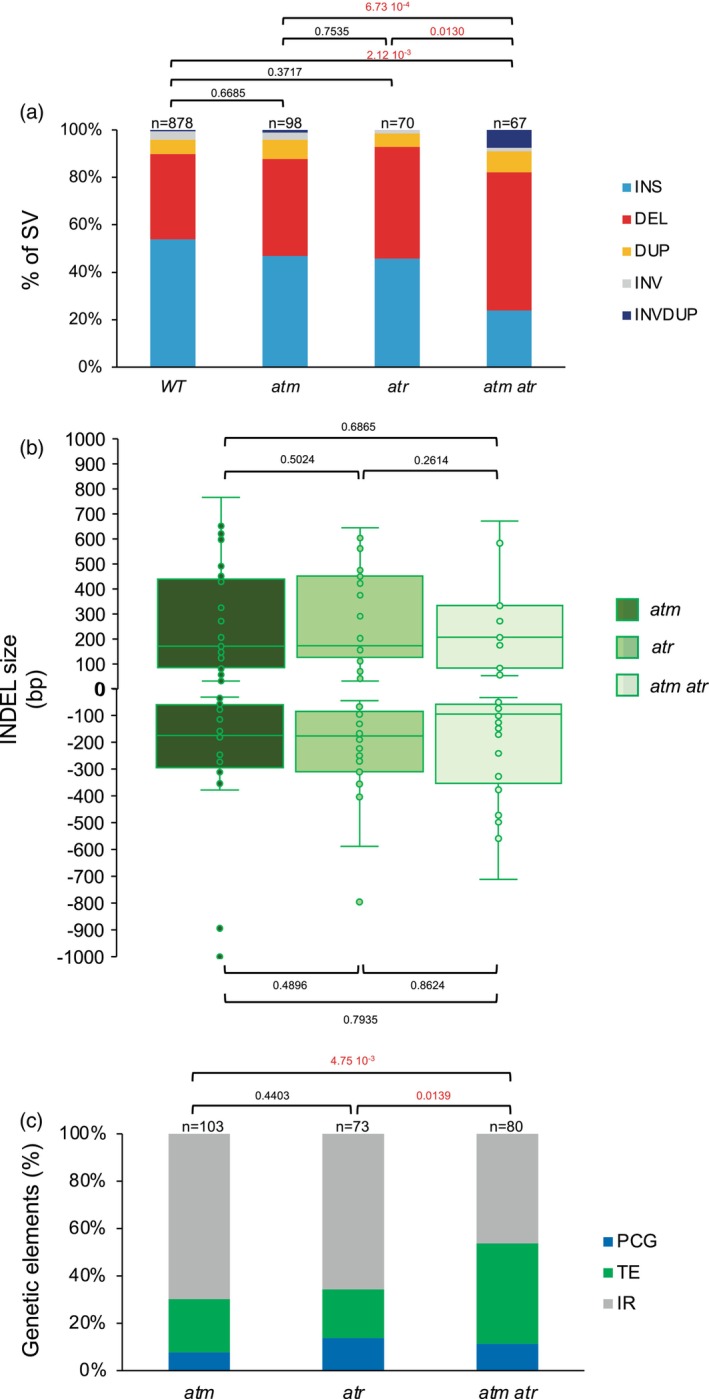
Characterization of the radiation‐induced genomic structural variations in *atm*, *atr* and *atm atr* Arabidopsis plants. (a) Histogram representing the distribution of the genomic SV identified in untreated WT Arabidopsis plants (relative to the TAIR 10 reference genome), *atm*, *atr*, and *atm atr* Arabidopsis plants. DEL, deletion; DUP, duplication; INS, insertion; INV, inversion; INVDUP, inversion duplication. *n* = total number of SV. Exact *P* values are shown (Chi‐squared test). (b) Box plots representing the INDELs sizes identified in *atm*, *atr*, and *atm atr* Arabidopsis plants. No significant differences have been found between genotypes (Mann‐Whitney Wilcoxon test). In boxplots, the central line and bounds of the box represent the median and the 25th and 75th quartiles, respectively. The whiskers represent 1.5× interquartile range of the lower or upper quartiles. (c) Histogram representing the distribution of the genetic elements (IR, intergenic regions; PCG, protein coding genes; TE, transposable elements) exhibiting SV in *atm*, *atr*, and *atm atr* Arabidopsis plants. Exact *P* values are shown (Chi‐squared test). *n* = total number of genetic elements containing SV.

We identified 98, 70 and 67 SV in untreated *atm*, *atr* and *atm atr* plants, respectively (Figure 6a). INDELS represent the most predominant types of SVs (Figure 6a). *atm atr* plants contain a larger proportion of deletions compared to each single mutant plants (Figure 6a), showing an additive effect of both mutations. Conversely, the size of INDELS do not significantly differ between single and double mutant plants (Figure 6b), suggesting that both ATM and ATR do not regulate repair mechanisms influencing INDELS lengths but rather different repair pathways leading to deletions. In *atm atr* plants, inversions‐duplications represent more than 7% of the SV (Figure 6a). This type of SV represents the common process controlling copy number variation (CNV; Schubert & Vu, 2016) and suggests that error‐free repair mechanisms may have been derepressed in these double mutant plants.

While, in single *atm* and *atr* mutant plants, IR represent the main genomic regions exhibiting SVs, TE are more affected in *atm atr* double mutant plants (Figure 6c). This might reflect a synergism between both PI3‐like kinases in the maintenance of genome integrity in particular genetic entities.

Around 75% of the SV identified in *atm* and *atr* plants occurred in pericentromeric regions, highlighting that this part of the genome is, like in WT plants, more prone to be rearranged (Figure S7a). This also suggests that factors likely other than ATM and ATR contribute to the maintenance of genome integrity in chromosomes arms.

In order to further investigate the role of ATM and ATR on genome stability, we decided to expose *atm* and *atr* plants to protons and UV‐B, respectively. We did not use *atm atr* plants in this experiment due to the low recovery rate of this double mutant. Protons irradiations form mainly DSB that are signaled by ATM (Shiloh, 2001) while UV‐B exposure induces the formation of photodamage interfering with transcription, replication, which is preferentially signaled by ATR (Shiloh, 2001). SV previously identified in untreated *atr* and *atm* plants have been subtracted to each corresponding treated mutant plants, in order to identify radiations‐induced SV (Figure 1d,e).

**Figure 7:**
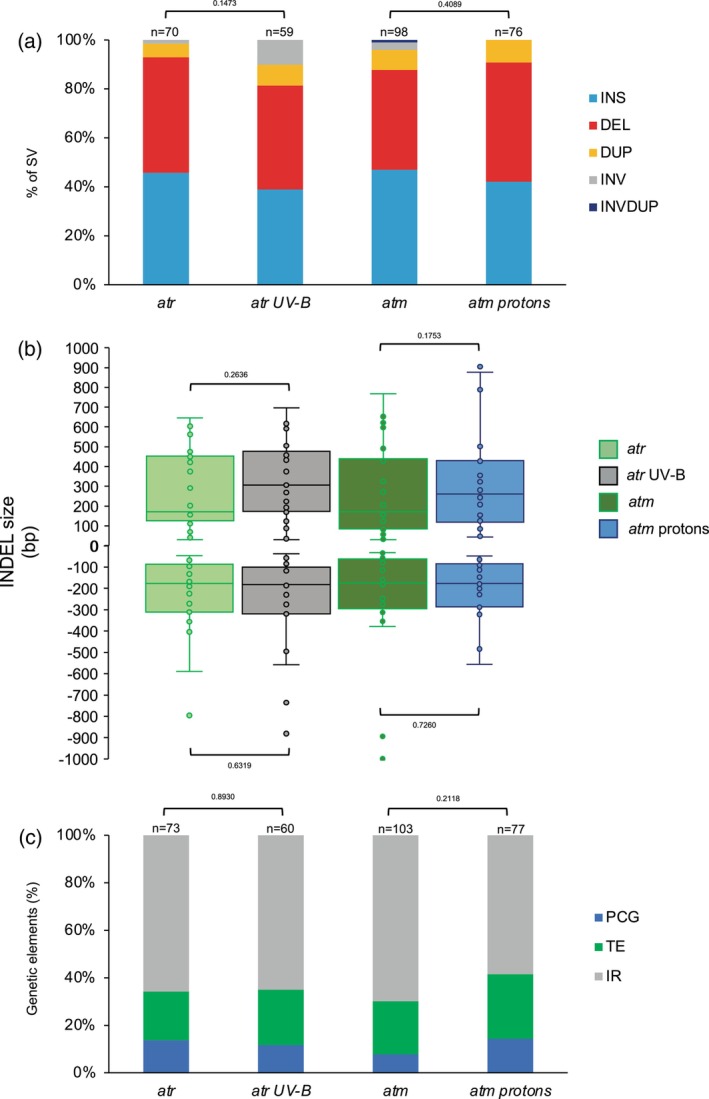
Characterization of the radiation‐induced genomic structural variations in *atr* and *atm* Arabidopsis plants. (a) Histogram representing the distribution of the genomic SV identified in *atr*, UV‐B‐treated *atr*, *atm* and protons‐treated *atm* Arabidopsis plants. DEL, deletion; DUP, duplication; INS, insertion; INV, inversion; INVDUP, inversion duplication. *n* = total number of SV. Exact *P* values are shown (Chi‐squared test). (b) Box plots representing the size of the INDELs identified in in *atr*, UV‐B‐treated *atr*, *atm* and protons‐treated *atm* Arabidopsis plants. No significant differences have been found between untreated and treated plants (Mann‐Whitney Wilcoxon test). In boxplots, the central line and bounds of the box represent the median and the 25th and 75th quartiles, respectively. The whiskers represent 1.5× interquartile range of the lower or upper quartiles. (c) Histogram representing the distribution of the genetic elements (IR, intergenic regions; PCG, protein coding genes; TE, transposable elements) exhibiting SV in *atr*, UV‐B‐treated *atr*, *atm*, and protons‐treated *atm* Arabidopsis plants. Exact *P* values are shown (Chi‐squared test). *n* = total number of genetic elements containing SV.

UV‐B and protons irradiations induced the formation of 59 and 76 SV in *atr* and *atm* plants, respectively (Figure 7a). Importantly, each treatment did not lead to a significant redistribution of SV types in both mutant plants (Figure 7a), showing that irradiations did not change the outcome of the repair processes. INDELS are the predominant SV formed and their sizes remain significantly unchanged between untreated and treated mutant plants (Figure 7b). Both TE and IR represent the majority of the genetic entities containing SV, which are located in the vicinity of chromocenters (Figure 7c; Figure S7b). These results suggest that, even upon exposure to radiations, ATM or ATR do not regulate repair pathways leading to the formation of a particular types of SV at specific genetic entities.

We also compared SV between WT‐ and mutant‐treated plants to uncover whether irradiation would reveal a particular role for these PI3‐like kinases in the protection of genomic regions/entities or against the formation of certain types of SVs. We found that protons‐treated *atm* plants exhibit more deletions and insertions, than WT‐irradiated plants (Figure S8a). This reveals that ATM mainly restricts INDELs formation upon protons exposure. Conversely, both UV‐B‐treated WT and *atr* plants display the same distribution of SVs (Figure S8b). Irradiation and absence of the PI3‐like kinases did not lead to a pronounced effect on genome integrity at particular genetic entities (Figure S8c,d). Surprisingly, protons exposure of *atm* plants leads to significant shorter deletions than WT‐treated plants (Figure S8e,f), highlighting that particular DSB repair mechanisms might have been derepressed in these mutant plants.

Altogether, these analyses show that ATM and/or ATR regulate genome integrity, mostly at TE‐IR, and that genic regions are less prone to be rearranged, even in the absence of these PI3‐like kinases. Thus, these results address the question of the putative existence of ATM‐, ATR‐independent DDR mechanisms acting in chromosomes arms (i.e., at PCG).

### Analysis of genomic regions flanking the deletions highlights homology‐directed DSB repair

**Figure 8:**
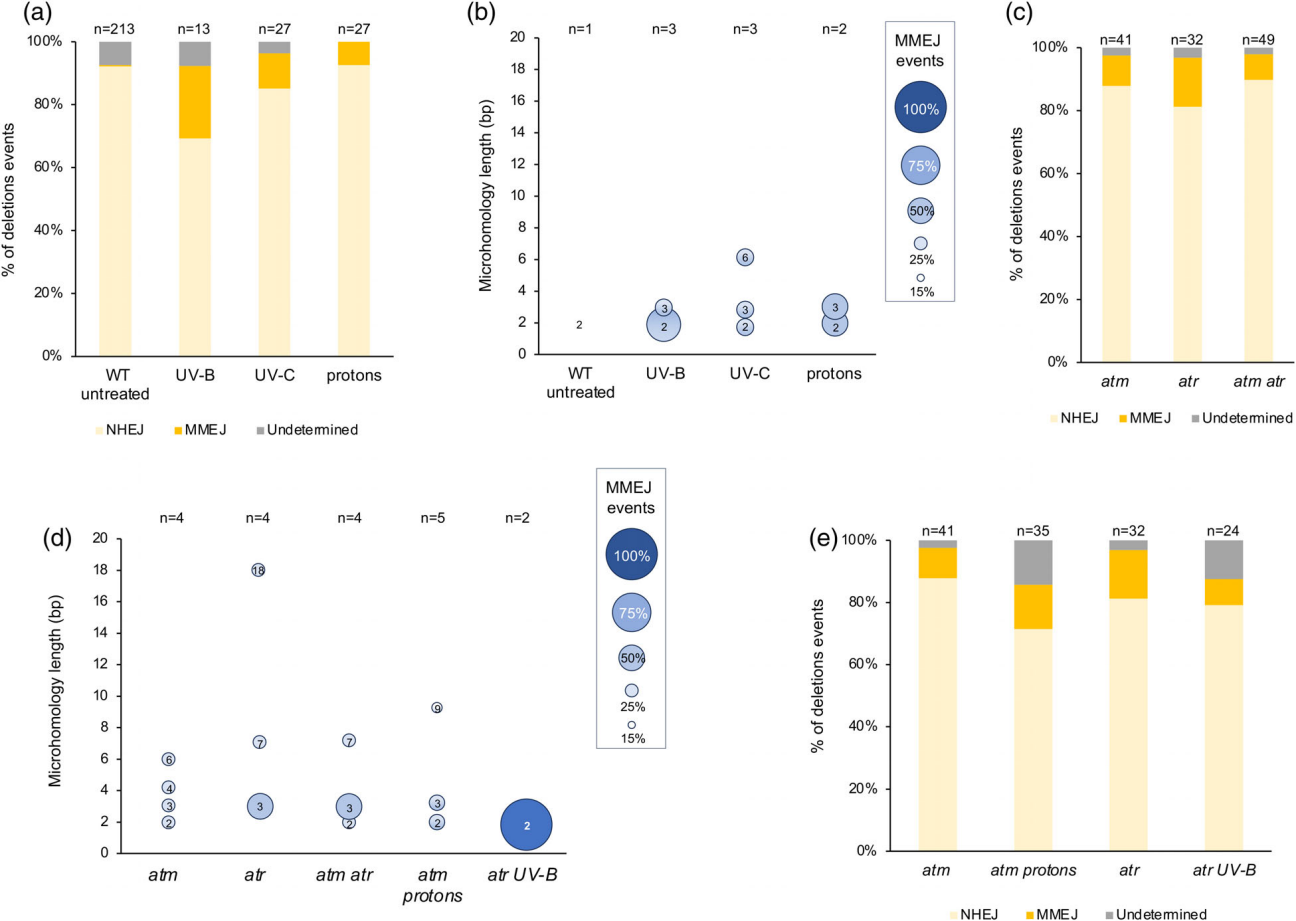
Characterization of end‐joining repair mechanisms. (a) Histogram representing the distribution of the NHEJ or MMEJ sequences signatures identified in the flanking regions of deletion in UV‐B‐, UV‐C‐, and protons‐treated WT Arabidopsis plants. *n* = total number of deletions. MMEJ, microhomology‐mediated end joining; NHEJ, non‐homologous end‐joining; Undetermined, low sequence quality in one of the flanking regions. (b) Bubble chart representing microhomologies lengths and their frequencies within MMEJ events identified upon UV‐B, UV‐C and protons irradiation of WT Arabidopsis plants. *n* = total number of MMEJ events. The size of the bubble corresponds to the percentage of MMEJ events for each microhomology length. Size (in bp) of the microhomology is indicated in each bubble. (c) Same as (a) for *atm*, *atr*, and *atm atr* plants. (d) Same as (b) for *atr*, UV‐B irradiated *atr*, *atm*, and protons‐irradiated *atm* plants. (e) Same as (a) for *atm*, *atr*, *atm atr*, UV‐B irradiated *atr*, and protons‐irradiated *atm* plants.

DSB are repaired by either NHEJ or HR pathways that are error prone and error free mechanisms, respectively (Puchta, 2005; Schuermann et al., 2005). Several mechanisms also use homologous sequences flanking the DSB to perform homology‐directed DSB repair (Puchta, 2005). Long reads sequencing data allowed determining to which extents, NHEJ and homology‐directed DSB repair have been used in our different experimental conditions. For this, we analyzed the flanking regions of the full set of deletions identified in WT‐treated Arabidopsis plants and in both untreated‐treated *ATM* and/or *ATR* deficient plants. Importantly, we mainly identified NHEJ patterns and 1 MMEJ event in WT untreated plants among the 213 deletions analyzed (Figure 8a). In WT‐irradiated plants, between 7.5 and 23% of the genomic regions, flanking directly the deletions, contain homologous sequences, suggesting that MMEJ repair pathway has been used (Figure 8a; Table S1). However, the source of irradiation did not significantly change the rate of NHEJ versus MMEJ (Figure 8a). The length of these micro homologies (2 and 6 bp) strongly suggests that the MMEJ pathway has been used to repair the UV‐induced DNA breaks and would rather rule out the use of the SSA pathway (Figure 8b; Table S1).

Single *atm* and *atr* mutant plants, as well as double *atm atr* mutant plants, exhibit between 8 and 15% of the sequences, flanking directly the deletion, with homologous repeats (Figure 8c,d; Table S1). They range from 2 to 18 bp in length, showing that MMEJ repair has been used (Figure 8c,d; Table S1). A median effect could be observed in *atm atr* plants compared to each single mutant plants (Figure 8c), suggesting that both PI3‐like kinases might prevent independently the use of the MMEJ repair pathway.

Upon exposure to protons and to UV‐B, *atm* and *atr* plants did not exhibit significant changes in the use of NHEJ and of MMEJ repair pathways (Figure 8e). The length of the micro homologies remains unchanged in *atm* treated plants compared to untreated plants (Figure 8d), suggesting that ATM plays a minor role in homology‐directed repair in response to ionizing radiations. Conversely, in UV‐B‐irradiated *atr* plants, we identified a decrease of the number of MMEJ events and of the length of the micro‐homologies (Figure 8d). This is likely that UV‐B exposure triggers the use of other repair pathways that quantitatively and qualitatively affect the MMEJ mechanism.

These results provide an overview of the balance between repair mechanisms (NHEJ and homology‐directed DSB repair), in WT and in DDR mutant plants, that have led to deletions upon different treatments.

## DISCUSSION

The use of long reads sequencing technology allowed characterizing the rearrangements of the linear genome structure of WT Arabidopsis plants and of DDR deficient plants (*atm* and *atr*) exposed to non‐ionizing (UV‐B and UV‐C) and ionizing radiations (protons). We identified that most of the radiations‐induced SVs occurred in heterochromatic regions in both WT and DDR deficient Arabidopsis plants. We found that the 2 PI3‐like protein kinases, ATM and ATR, restrict INDELs formation. We determined that deletions are the outcome of NHEJ and homology‐directed DSB repair pathways, and identified that ATM and ATR repress the MMEJ repair pathway.

A crucial step in such comparative genomic approach was to quantify the putative variability that may contain WT Col‐0 Arabidopsis plants, in order to define our reference genome and to identify radiations‐induced SVs. We found, in three independent biological replicates, more than 800 SVs compared to the TAIR10 reference genome with a core of 138 SVs. The improvement of the sequencing resolution was already validated with the Col‐*CEN Arabidopsis thaliana* genome assembly that recently resolved all five centromeres (Naish et al., 2021). This holds true for the accurate assembly of others types repetitive sequences, TEs and small INDELS (Lian et al., 2024; Naish et al., 2021). Importantly, the numerous SVs identified in our population of WT Col‐0 plants, may have occurred during plant development in cells giving rise to the germline and/or during meiosis. In addition, the heterogeneity of the somatic plant cells (i.e., leaves) could have contributed to the large number of SV found in the three independent biological replicates. Indeed, it has been reported that independent Arabidopsis reporter lines harboring a homologous recombination substrate exhibit different recombination rates in control conditions and also upon exposure to genotoxic stresses (Molinier et al., 2004; Swoboda et al., 1994). This highlights that DSB formation and repair efficiency vary between genomic regions, plant material and plant species.

We identified that most of the radiations‐induced SV occurred within constitutive heterochromatin, suggesting that this part of the genome tends to be more prone to be rearranged than euchromatic regions (Lian et al., 2024). It was demonstrated that CRISPR–Cas9‐induced SV, as well as the repair outcomes, are highly influenced by chromatin features (Filler‐Hayut et al., 2021; Weiss et al., 2022). Repressive chromatin landscape (i.e., high DNA methylation) reduces CRISPR–Cas9 mutagenesis efficiency (Weiss et al., 2022). The discrepancy between the frequency of radiations‐ and CRISPR‐Cas9‐induced SVs in heterochromatin might reflect different damaging and/or repair efficacies. Constitutive heterochromatin contains high amounts of repeats that are source of homologies for repair (Avramova, 2002; Orel et al., 2003; Schmidt & Anderson, 2006). Indeed, DSB formed in repeats often leads to chromosomal rearrangements (Pâques et al., 1998). For example, centromeric regions containing 180 bp repeats, display high structural dynamics (Naish & Henderson, 2024). CNV of 45S rDNA has been characterized upon CRISPR‐Cas9‐induced DSBs (Hacker et al., 2022; Lopez et al., 2021). Moreover, SVs in repeats are likely less deleterious than rearrangements occurring in single or low copies PCG. Our observation is in agreement with the recent study showing that the CRISPR‐Cas9 induction of DSB, in different tomato PCG, leads predominantly to a precise repair (Ben‐Tov et al., 2024). Thus, genome surveillance efficiency differs between euchromatin and heterochromatin, due to the presence of repetitive sequences and, likely, to others unknown features.

The formation of higher amounts of DNA damage, the activation of specific DNA repair (sub)pathways or the presence of particular repair intermediates could also influence the formation of SV. Indeed, UV‐induced photodamage are enriched in constitutive heterochromatin (Graindorge et al., 2019; Johann to Berens et al., 2023) which are predominantly processed by excision repair (i.e., Global Genome Repair; Molinier, 2017). This slow DNA repair mechanism could favor the presence of repair intermediates that likely become substrates for rearrangements (Schärer, 2013). In euchromatin, hypo‐mutation was demonstrated to be associated with H3K4me1‐rich gene bodies and essential genes (Monroe et al., 2022; Quiroz et al., 2024) whereas T‐DNA and TE insertions occur preferentially in PCG and in their downstream regions, respectively (Brunaud et al., 2002; Sigman & Slotkin, 2016; Zhang et al., 2023). Upon UV‐B exposure, both G:C → A:T transition rate (Willing et al., 2016) and SV are enhanced in PCG (this study). Thus, euchromatin exhibits different behaviors against point mutations and SVs (i.e., insertions), suggesting that the epigenome‐recruitment of DNA repair factors is complex and tightly regulated.

ATM and ATR are master regulators of the DDR (Shiloh, 2001). Long reads sequencing technology offered the possibility to study, with an improved resolution, the roles of ATM and ATR in the maintenance of genome integrity. We found that in absence of genotoxic stress, each kinase restricts the formation of INDELs and that the number of SV did not significantly change in *atm atr* double mutant plants. These SV occurred predominantly in TE and IR, highlighting that PCG remained efficiently protected from rearrangements despite the absence of these key kinases. This suggests that either DSB occur preferentially in heterochromatin, or that others key regulatory factors trigger the DDR in genic regions in an ATM−/ATR‐independent manner.

The analysis of both inserted and deleted regions allowed characterizing the origins of these sequences and the type of the repair pathway used, respectively. Transposition events (Debladis et al., 2017; Mirouze et al., 2009), extra chromosomal circular DNA (ecc DNA; Ito et al., 2011) and capture of DNA sequences from distant genomic regions (Gorbunova & Levy, 1997) can lead to neo insertions into the genome. For example, in *A. thaliana*, the *Ty1*/*Copia*‐like retrotransposon *ONSEN*, could be mobilized upon heat stress exposure (Ito et al., 2011) and *de novo* integrations of TE‐derived eccDNA have been characterized in genic regions (Zhang et al., 2023). Such neo‐insertions could lead to change in expression of neighboring PCG (Roquis et al., 2021; Thieme et al., 2022) and has been shown to be a major source of genetic variation in *A. thaliana* (Baduel et al., 2021). However, we only found truncated TE, in both WT and DDR deficient plants, suggesting that our growth conditions did not favor *per se* transposition.

During NHEJ repair, genomic sequences of different lengths and distant from the break point can be copied by synthesis‐dependent strand annealing like mechanisms (Gorbunova & Levy, 1997, 1999; Rubin & Levy, 1997; Salomon & Puchta, 1998). We found a preference for the intrachromosomal origin of the inserted sequences, supporting the idea that genomic regions in the vicinity of the DNA breaks are used as templates. This is likely that linear 1D organization and/or 3D genome folding favor such preferential use of intramolecular templates. Thus, these genomic features should be considered as parameter that could influence template availability during DSB repair.

Repeats/TE‐ (Cohen et al., 2008; Lanciano et al., 2021) or intramolecular recombination‐derived eccDNA (Molinier et al., 2004; Peterhans et al., 1990) are unstable episomes that can reintegrate and lead to SV (Peng et al., 2022). Arabidopsis plants with altered methylome and/or silencing machinery exhibited accumulation of TE‐derived eccDNA and neo‐insertions (Zhang et al., 2023). Intramolecular recombination events produce ecc repair intermediates that have been shown to reintegrate into the genome and to form SV (Molinier et al., 2004). Thus, different origins of episomes exist and are source of genetic variability.

The loss of genetic information also occurs upon DSB repair. Indeed, deletions are the outcome of the NHEJ or of homology‐directed repair pathways (Puchta, 2005; Schubert & Vu, 2016). Nevertheless, it is important to notice that some NHEJ events can also be associated with filler DNA insertions (Gorbunova & Levy, 1997). Vu et al. (2017) showed that deletions occurred mainly via SSA and NHEJ. Repair events, leading to deletions, are mainly due to NHEJ, if long homologous sequences are missing in the vicinity of the break. Nevertheless, short homologies (between 2 and 25 bp), could be used by the MMEJ pathway. NEHJ patterns represent the main outcome of repair and MMEJ events have been identified only in irradiated and in DDR deficient plants. Although, homology‐directed repair pathways (SSA, MMEJ) have been described to be the main DSB repair pathways used in higher eukaryotes (Puchta, 2005), we did not identify canonical SSA events with 20–25 bp micro‐homologies. This could be likely due to the type of induced DNA damage and/or to their genomic locations. Indeed, the availability of particular repair factors, as well as the nucleotide context, could favor the use of one or others pathways (Ceccaldi et al., 2016). MMEJ acts in DSB repair in yeast, mammals (McVey & Lee, 2008) and in plants (Heacock et al., 2004). Moreover, MMEJ seems to play an important role during the CRISPR/Cas‐based genome editing (Ata et al., 2018; Tan et al., 2020; Weiss et al., 2020). A synthesis‐dependent MMEJ (SD‐MMEJ) mechanism was also described and relies on *de novo* DNA synthesis to create microhomology (Yu & McVey, 2010). Plant organellar DNA polymerases have been shown to repair DSB by MMEJ (García‐Medel et al., 2019), suggesting that a SD‐MMEJ‐like mechanism exists in plants.

Using third generation sequencing technology we documented the effect of genotoxic stresses exposure on the whole linear genome structure, shedding the light on the stability of euchromatin versus the pronounced flexibility of heterochromatin. This experimental set‐up and the produced resources, open new perspectives to further study the damaging effect of particular genotoxic agents and the type of DNA repair processes used within genome and epigenome complexity.

## EXPERIMENTAL PROCEDURES

### Plant material and growth conditions

*Arabidopsis thaliana* ecotype Col‐0 was obtained from the Arabidopsis Biological Resource Stock Center (ABRC, Nottingham, UK; ID N1092). Plants were cultivated in soil in a culture chamber under a 16 h light (light intensity ∼150 μmol m^−2^ sec^−1^; 21°C) and 8 h dark (19°C) photoperiod. *A. thaliana atm‐2* and *atr*‐2 (Vespa et al., 2005) plants (Col‐0 ecotype) were also used. The progeny of *ATM* +/− plants was genotyped to recover all *ATM* −/− plants used in this study. *ATR* −/− plants is the 3rd generation of selfed *ATR* −/− plants. Double *atm atr* mutant plants were obtained by the crossing of *ATM* +/− plants with *ATR* −/− plants. F2 plants were genotyped to retrieve double *atm atr* mutant plants. Eight *atm atr* plants have been used for long reads sequencing experiments.

### Protons irradiations

Soil‐gown 21‐day‐old Arabidopsis Col‐0 plants (WT or *atm*) were exposed to a dose of 100 Gy (J/kg) of protons delivered by the Cyrce Cyclotron at IPHC (https://cyrce.fr/en/home/, TR24 cyclotron) with a proton beam energy of 25 MeV. At this energy, protons pass completely through the leaf (dose‐rate of 0.55 Gy/s). Five leaves from 20 plants were irradiated on a zone of 5 mm of diameter. Then 24 h upon exposure, the irradiated zone was cut with a hollow punch (5 mm) and snap frozen in liquid nitrogen. Leaves discs of twice 20 untreated plants have been harvested from the 2 independent biological replicates and pooled. This untreated plant material corresponds to replicate 1 for the determination of our WT Col‐0 pedigree.

### UV‐B irradiation

Soil‐gown 21‐day‐old Arabidopsis Col‐0 plants (WT or *atr*) were exposed during 15 min to four bulbs of UV‐B Broadband (Philips—TL 40W/12 RS SLV/25) to deliver a total dose of 4500 J/m^2^. Five leaves from 10 irradiated plants were harvested after 24 h upon irradiation and snap frozen in liquid nitrogen. Two independent biological replicates have been performed and pooled for long reads sequencing experiments. Five leaves twice from 10 untreated plants were harvested from the two independent biological replicates and pooled. This untreated plant material corresponds to replicate 2 for the determination of our WT Col‐0 pedigree.

### UV‐C irradiation

Soil‐gown 21‐day‐old Arabidopsis Col‐0 plants were exposed to 2000 J/m^2^ of UV‐C using the Stratalinker 2400 (Stratagene). Five leaves from 10 irradiated plants were harvested after 24 h upon irradiation and snap frozen in liquid nitrogen. Two independent biological replicates were performed and pooled for long reads sequencing experiments. Five leaves twice from 10 untreated plants were harvested from the two independent biological replicates and pooled. This untreated plant material corresponds to replicate 3 for the determination of our WT Col‐0 pedigree.

### Genomic DNA extraction and library preparation

Genomic DNA was prepared from 100 to 200 mg of leaves using the plant DNA extraction kit Nucleon Phytopure (Cytiva). RNaseA/T1treatment was performed and DNA was cleaned up using the MaXtract High Density kit (Qiagen) to recover high molecular weight DNA (HMW). Ultra‐long DNA library for Nanopore sequencing was produced from 100 to 200 fmoles of HMW genomic DNA using the NEBNext companion module (NEB) and the Ligation Native Sequencing Kit V9 (ONT). 5–50 fmoles of the library was loaded onto ONT FLO‐MIN R9.4.1 or ONT FLO‐PRO R9.4.1 R9.4.1 flow cells.

### Identification of genomic structural variants

Reads were sequenced on ONT FLO‐MIN R9.4.1 or ONT FLO‐PRO R9.4.1 R9.4.1 flow cells and basecalled with ont‐guppy‐gpu_6.3.8 with the model dna_r9.4.1_450bps_sup.cfg (Table S2). The analysis was performed using a Snakemake script adapted from the Oxford Nanopore Structural Variant pipeline (https://github.com/nanoporetech/pipeline‐structural‐variation). Sequencing quality was evaluated with MinIONQC (V 1.33.5). The mapping was performed with Minimap2 (V 2.17) using the *A. thaliana*, Col0‐TAIR10, as reference genome. The mapping quality was checked with Nanoplot (V 1.30.0). Coverage was evaluated by mosdepth (V 0.2.7) and Sniffles (V 1.0.11) was used to identify the structural variations (SV) in comparison with the reference genome Col0‐TAIR10. SVs have been filtered using the following parameters: minimal SV length 1 bp, maximal SV length 1 000 000 bp, minimal read length 1000 bp and minimal mapping quality 20.

SV of the same type (insertion, deletion, duplication, inversion or inversion duplication) with the same genomic coordinates (Chr, start‐end) ± 50 bp have been considered as identical.

### Characterization of the origins of insertions

Inserted sequences have been blasted using ncbi‐blast+ (blastn fonction) and annotated to retrieve the origin of each insertion.

### Characterization of NHEJ and MMEJ repair events

The flanking sequences (±50 bp) of the deleted regions have been fetched using samtools (command samtools faidx). Microhomologies have been manually curated to determine the rate of MMEJ events.

## AUTHOR CONTRIBUTIONS

SOS performed plant work and the bioinformatic analyses; AA performed the long reads sequencing; SS performed the plant work and the UV irradiations; SG set up the bioinformatic analysis; MP, JS, and CG performed the protons irradiations; QR and MR obtained the fundings, set–up, and performed the protons irradiations; JM designed experiments, obtained the fundings and wrote the manuscript.

## CONFLICT OF INTEREST

We declare no competing financial interests.

## Supporting information


**Figure S1.** TE superfamilies exhibiting SV in WT Arabidopsis plants.
**Figure S2.** TE superfamilies exhibiting SV in radiation‐treated WT Arabidopsis plants.
**Figure S3.** INDEL size in genetic elements of radiation‐treated WT Arabidopsis plants.
**Figure S4.** Origins of insertions in WT‐irradiated Arabidopsis plants.
**Figure S5.** Origins of insertions in radiation‐treated WT Arabidopsis plants.
**Figure S6.** Genomic SV overlapping with HOT regions.
**Figure S7.** Genomic locations of structural variations.
**Figure S8.** Comparisons of the radiation‐induced genomic structural variations between WT, *atr* and *atm* Arabidopsis plants.

**Table S1.** MMEJ events.

**Table S2.** Sequencing statistics.

## Data Availability

The fast5 files will be available on demand.
