# Supplementary material for: Characterization of radiations‐induced genomic structural variations in Arabidopsis thaliana
Source: Plant J. 2024 Dec 1;121(1):e17180. doi: 10.1111/tpj.17180 (PMC11712536; doi:10.1111/tpj.17180)
Supplement: Supplementary file 1 — Figure S1. TE superfamilies exhibiting SV in WT Arabidopsis plants. Figure S2. TE superfamilies exhibiting SV in radiation‐treated WT Arabidopsis plants. Figure S3. INDEL size in genetic elements of radiation‐treated WT Arabidopsis plants. Figure S4. Origins of insertions in WT‐irradiated Arabidopsis plants. Figure S5. Origins of insertions in radiation‐treated WT Arabidopsis plants. Figure S6. Genomic SV overlapping with HOT regions. Figure S7. Genomic locations of structural variations. Figure S8. Comparisons of the radiation‐induced genomic structural variations between WT, atr and atm Arabidopsis plants. [file TPJ-121-0-s001.zip › SUPPORTING INFORMATION legends.pdf]

SUPPORTING INFORMATION

SUPPORTING FIGURES

Figure S1: TE superfamilies exhibiting SV in WT arabidopsis plants.
Histogram representing the distribution of TE superfamilies exhibiting SV identified in the 3 independent biological replicates of WT (Col-0) arabidopsis plants. Exact p values are shown (Chi square test).

Figure S2: TE superfamilies exhibiting SV in radiation-treated WT arabidopsis plants.
Histogram representing the distribution of TE superfamilies exhibiting SV identified in WT Arabidopsis plants treated with either UV-B, UV-C or protons. Exact p values are shown (Chi square test).

Figure S3: INDEL size in genetic elements of radiation-treated WT arabidopsis plants.
a. Box plots representing the INDELs sizes identified in Protein Coding Genes: PCG; Transposable Elements: TE and Intergenic regions: IR. Exact p values are shown (Mann Whitney Wilcoxon test). na: non-applicable. In boxplots, the central line and bounds of the box represent the median and the 25th and 75th quartiles, respectively. The whiskers represent 1.5× interquartile range of the lower or upper quartiles. **b.** Histogram representing the distribution of SV (INS: Insertion; DEL: Deletion; DUP: Duplication; INV: Inversion; INVDUP: Inversion Duplication) in PCG, TE and IR. n= total number of SV in genetic elements.

Figure S4: Origins of insertions in WT-irradiated Arabidopsis plants.
a. Histogram representing the origin of the insertions (Protein Coding Genes: PCG; Transposable Elements: TE and Intergenic regions: IR) identified in WT Arabidopsis plants exposed to UV-B, UV-C or protons. **b.** Histogram representing the origin of the insertions identified in PCG of WT Arabidopsis plants exposed to UV-B, UV-C or protons. na: non-applicable. **c.** Same as **b.** for TE. **d.** Same as **b.** for IR. n= total number of insertions in genetic elements.

Figure S5: Origins of insertions in radiation-treated WT Arabidopsis plants.
Circos representation of the origins of insertions in WT Arabidopsis plants treated with either UV-B, UV-C or protons. Black rectangles represent the centromeres.

Figure S6: Genomic SV overlapping with HOT regions.

a. Venn diagram representing the overlap of SV identified in irradiated WT Arabidopsis plants and hotspots of rearrangements (HOT; Jiao and Schneeberger, 2020). **b.** Circos representation of genomic SV of irradiated WT Arabidopsis plants overlapping with HOT. Black rectangles represent the centromeres.

Figure S7: Genomic locations of structural variations.

a. Circos representation of genomic SV (INS: Insertion; DEL: Deletion; DUP: Duplication; INV: Inversion; INVDUP: Inversion Duplication) identified in *atm*, *atr* and *atm atr* plants. **b.** Same as **a.** for *atr*, UV-B irradiated *atr*, *atm* and protons-irradiated *atm* plants. Black rectangles represent the centromeres.

Figure S8: Comparisons of the radiation-induced genomic structural variations between WT, *atr* and *atm* Arabidopsis plants.

a. Histogram representing the distribution of the different types of genomic SV identified in WT and *atm* plants irradiated with protons. INS: Insertion; DEL: Deletion; DUP: Duplication; INV: Inversion; INVDUP: Inversion Duplication. n= total number of SV. Exact p values are shown (Chi square test). **b.** Same as **a.** for WT and *atr* plants irradiated with UV-B. **c.** Histogram representing the distribution of the genetic elements (Protein Coding Genes: PCG; Transposable Elements: TE and Intergenic regions: IR) exhibiting SV in WT and *atm* plants irradiated with protons. Exact p values are shown (Chi square test). n= total number of SV in genetic elements. **d.** Same as **c.** for WT and *atr* plants irradiated with UV-B. **e.** Box plots representing the size of the INDELs identified in WT and *atm* plants irradiated with protons. Exact p values are shown (Mann Whitney Wilcoxon test). **f.** Same as **e.** for WT and *atr* plants irradiated with UV-B.

SUPPORTING TABLES

Table S1: MMEJ events

Table S2: Sequencing statistics
