# Supplementary figures and images for: Characterization of radiations‐induced genomic structural variations in Arabidopsis thaliana
Source: Plant J. 2024 Dec 1;121(1):e17180. doi: 10.1111/tpj.17180 (PMC11712536; doi:10.1111/tpj.17180)

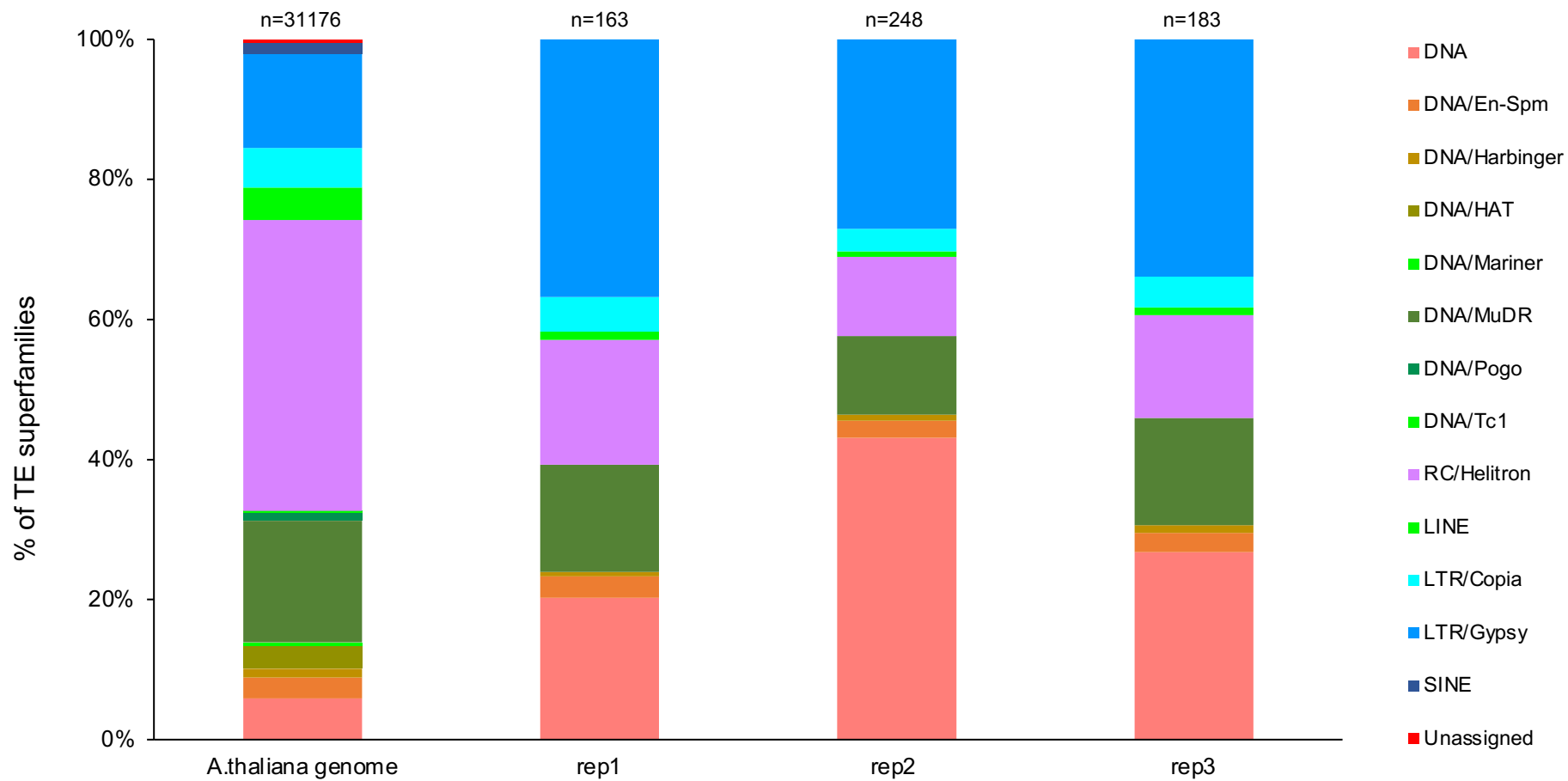

Figure S1

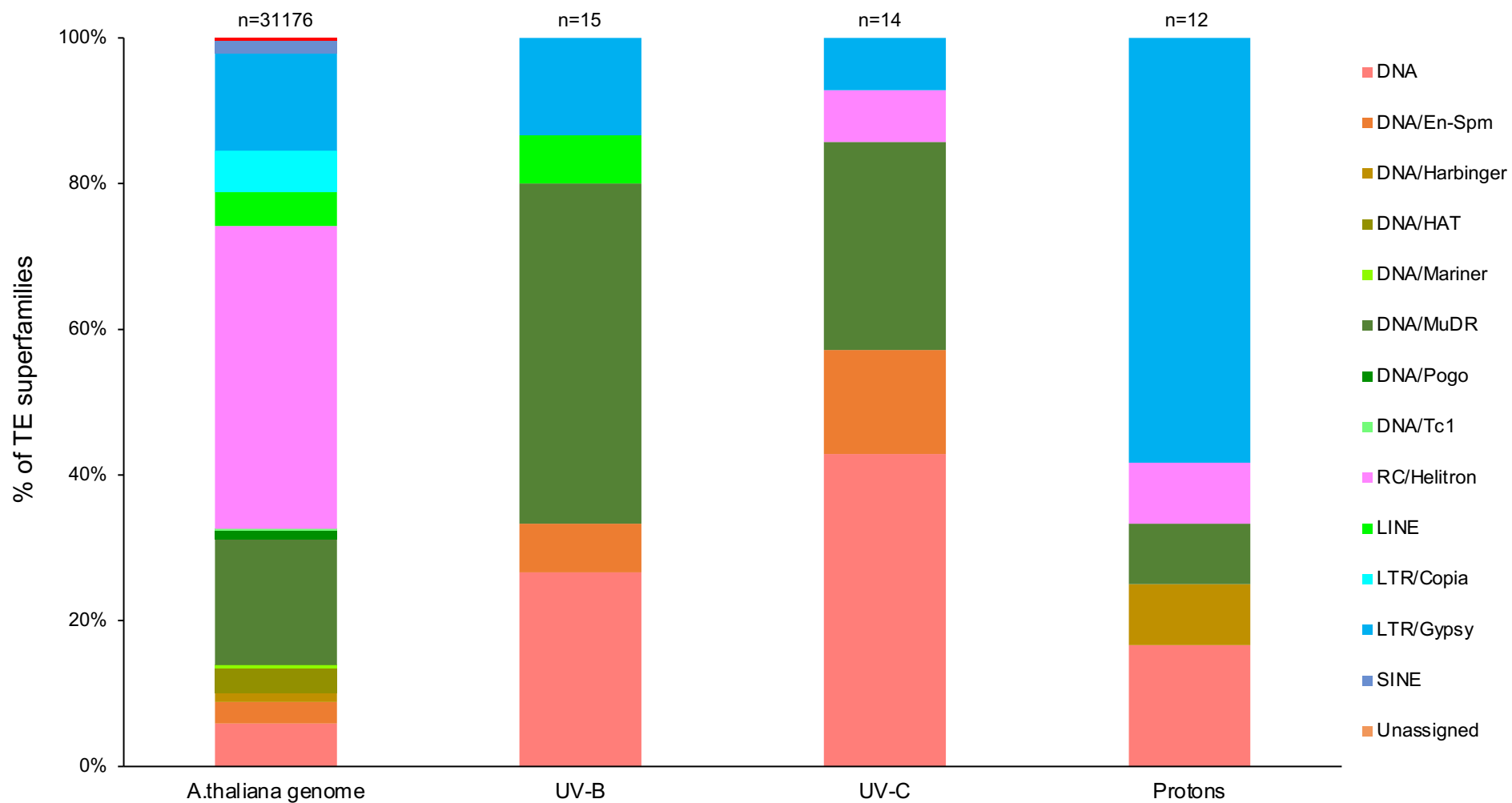

Figure S2

**a**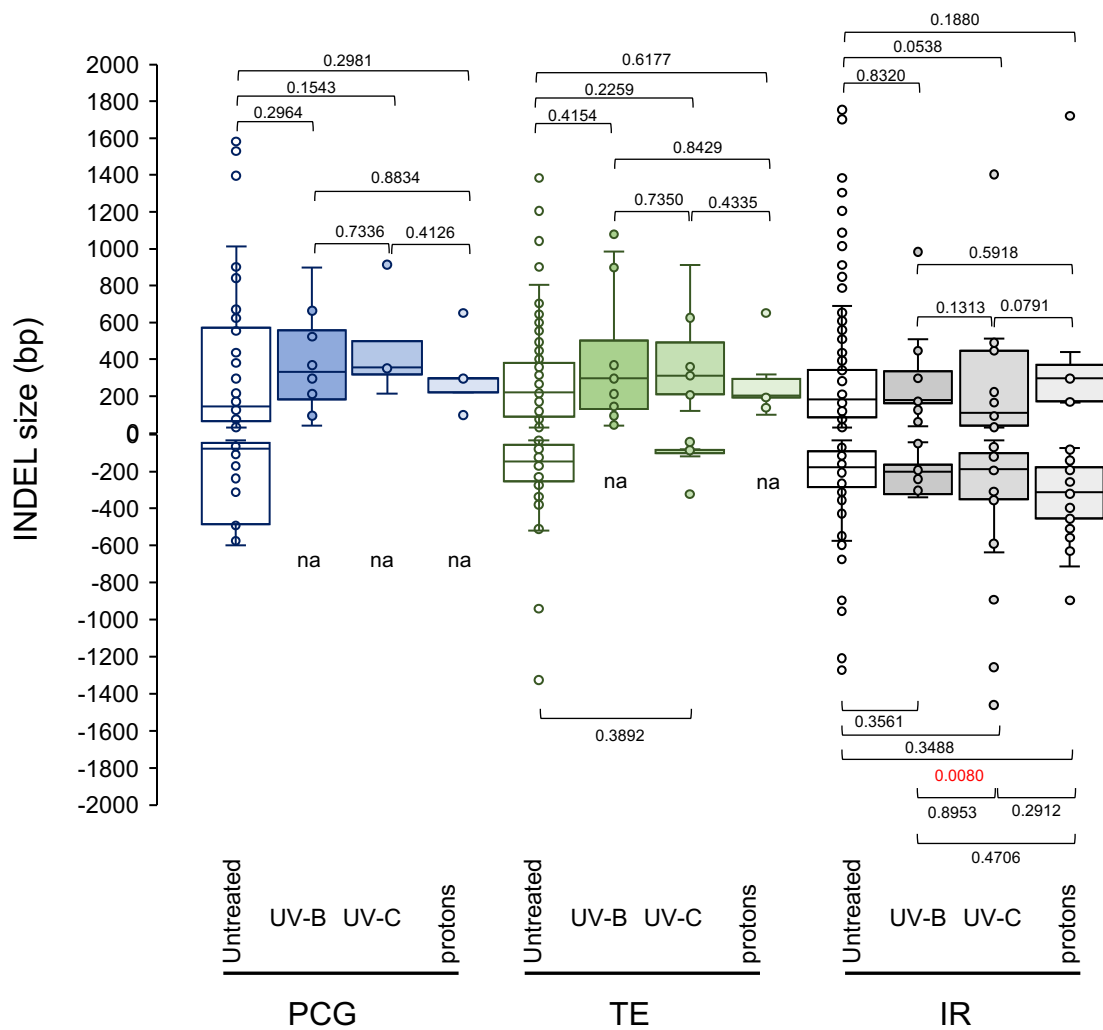**b**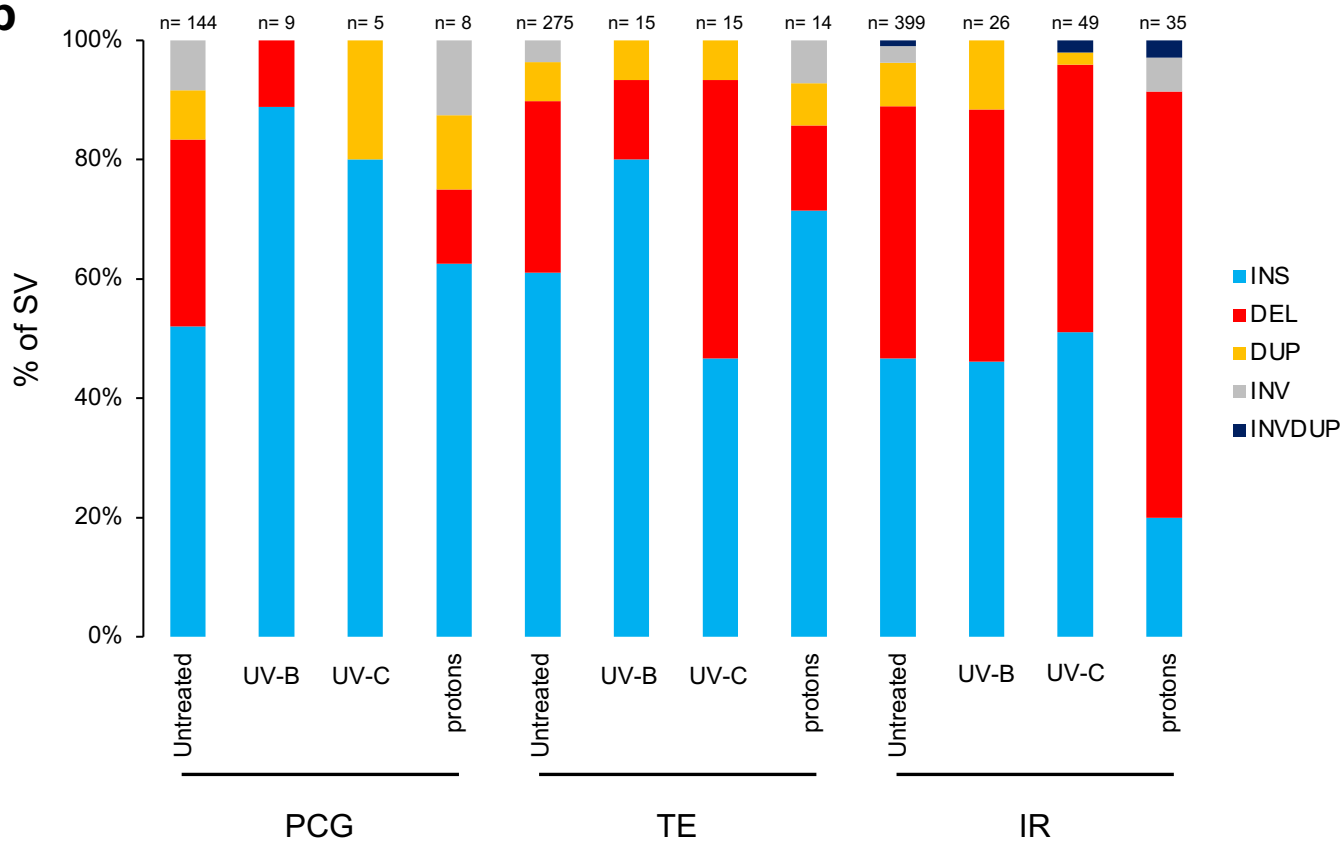

Figure S3

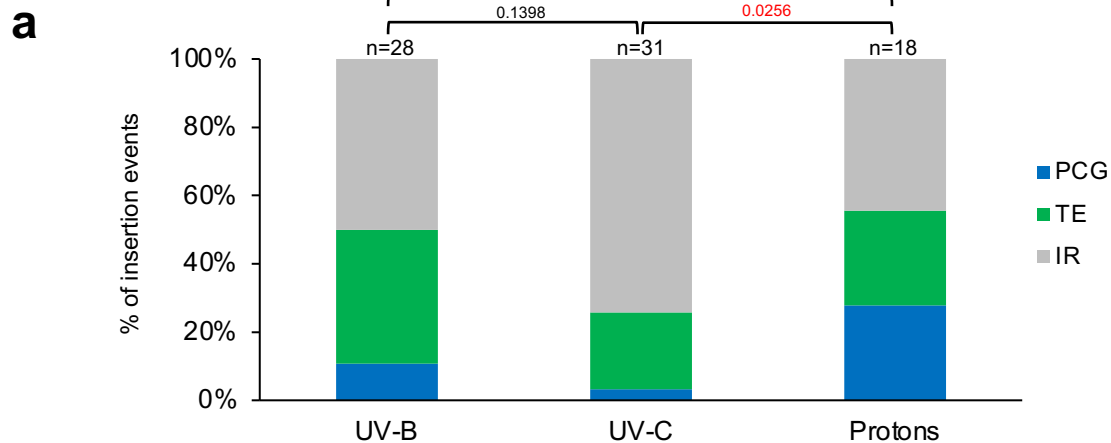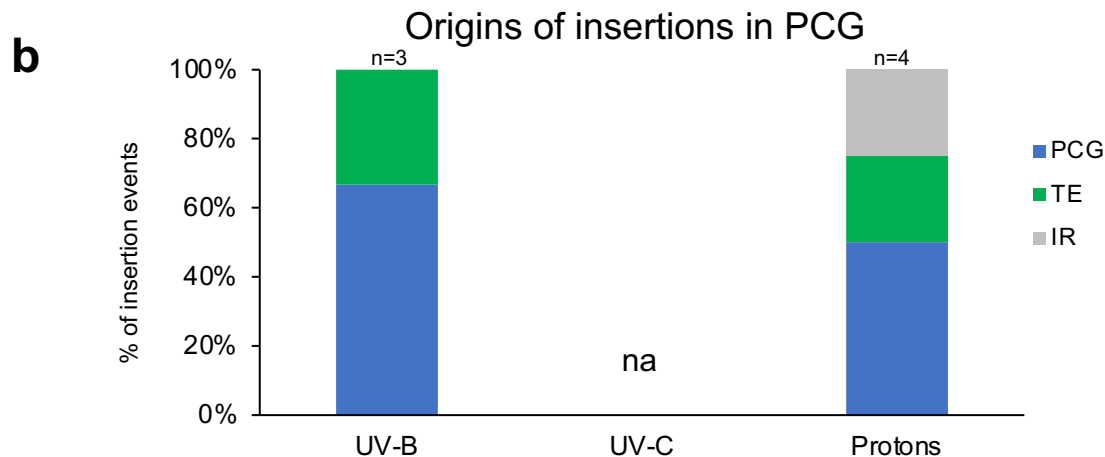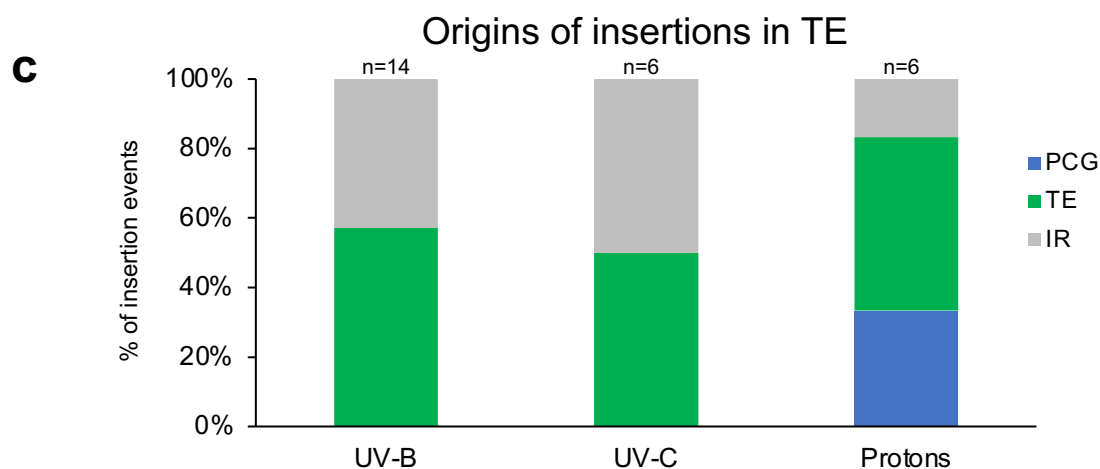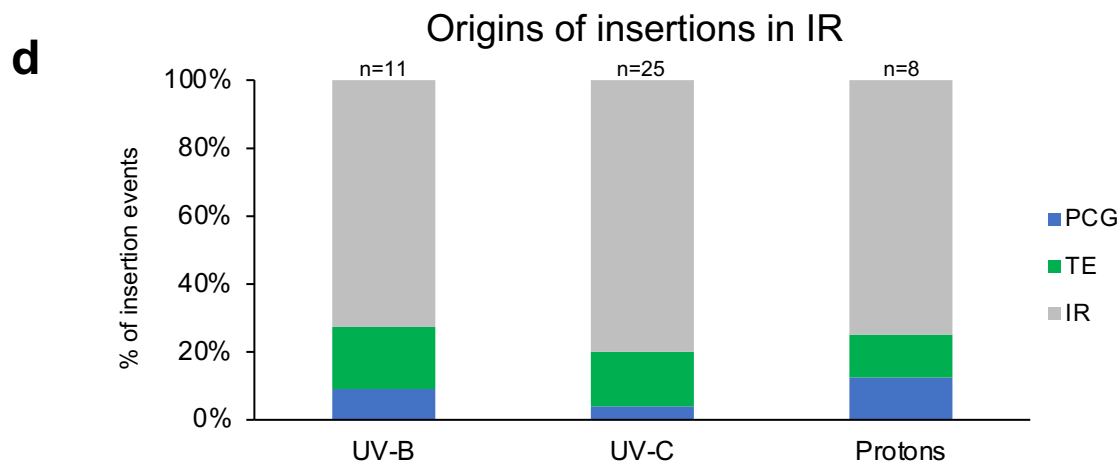

Figure S4

UV-B

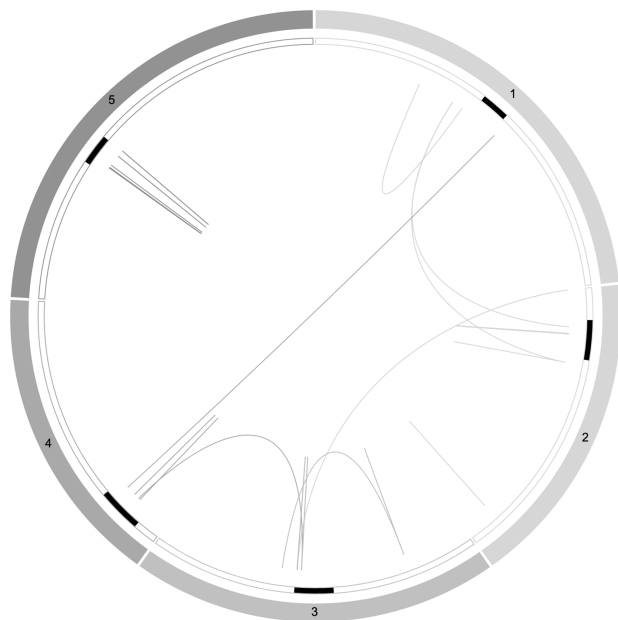

UV-C

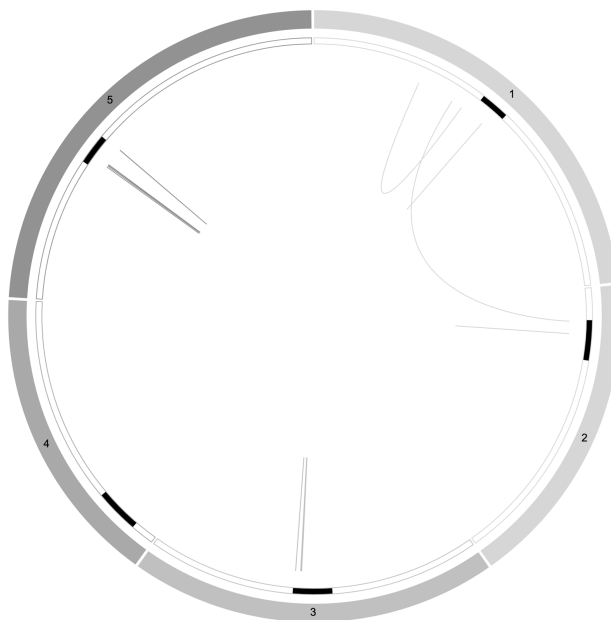

Protons

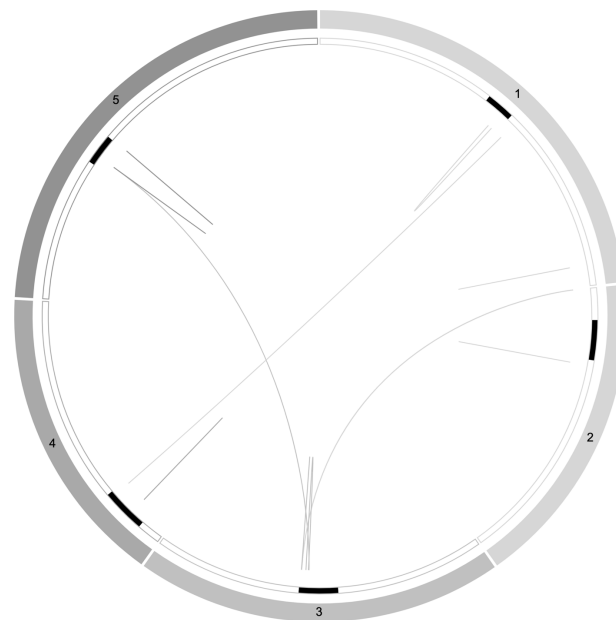

**a**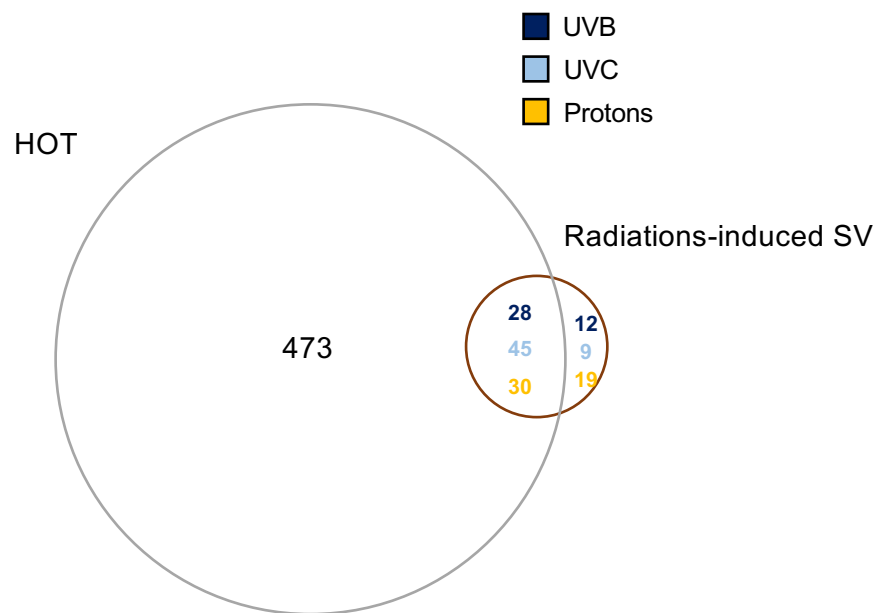**b**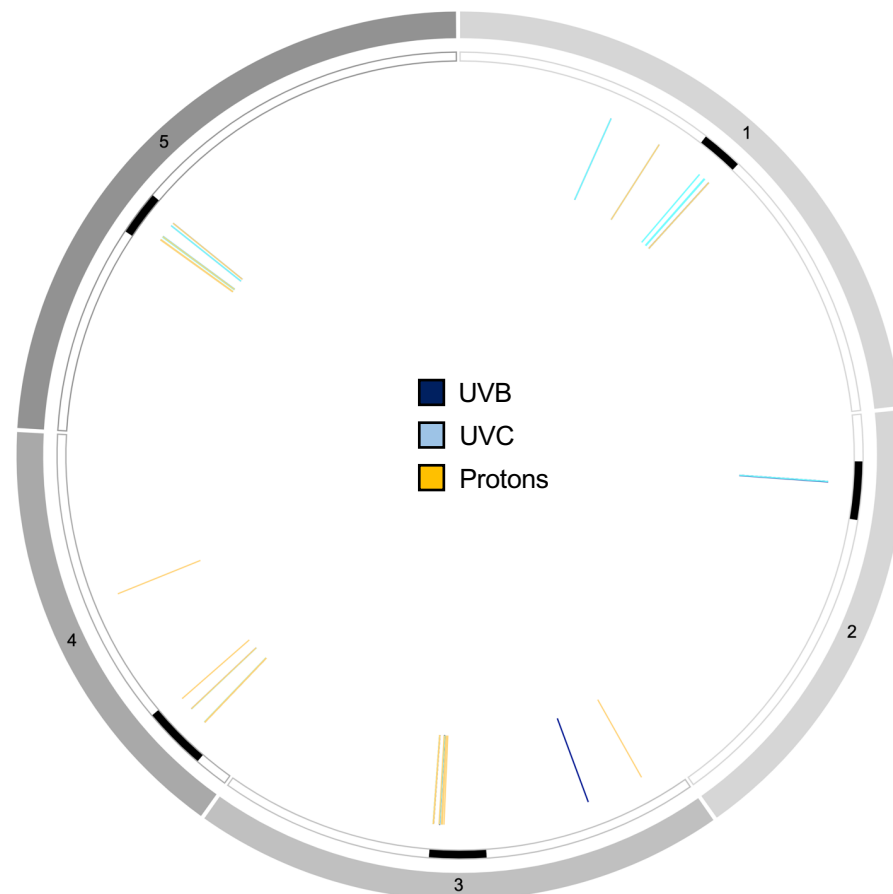

**a**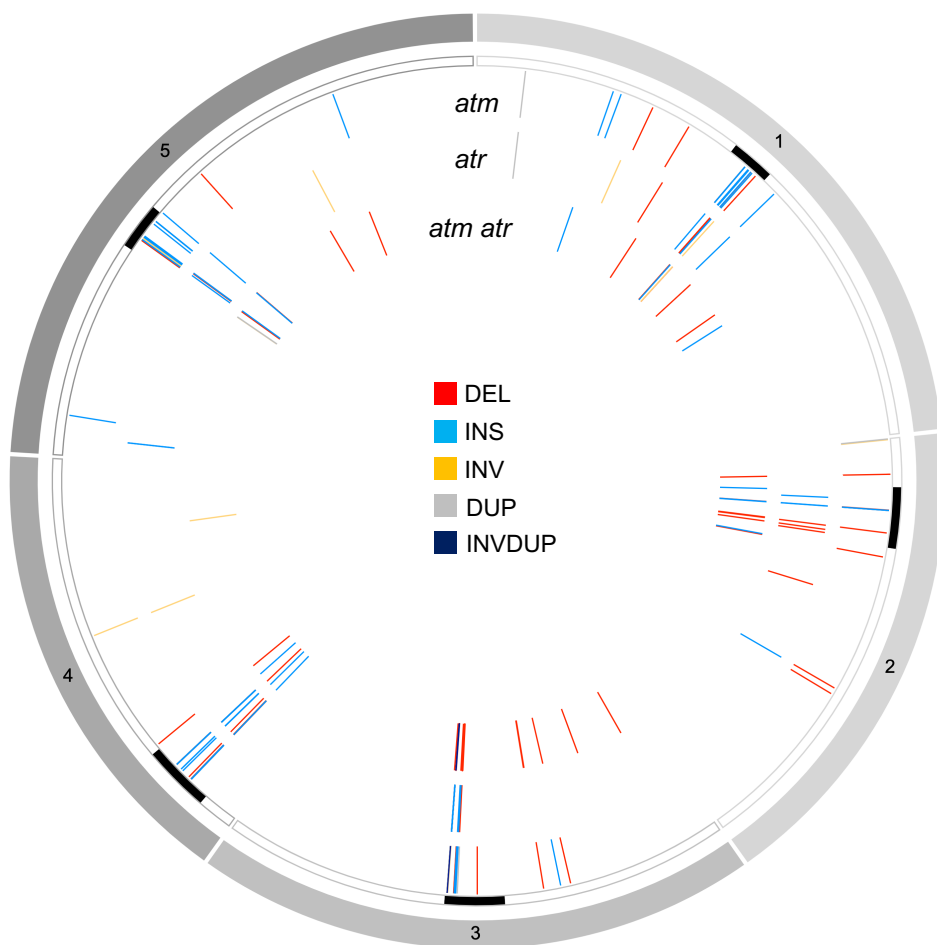**b**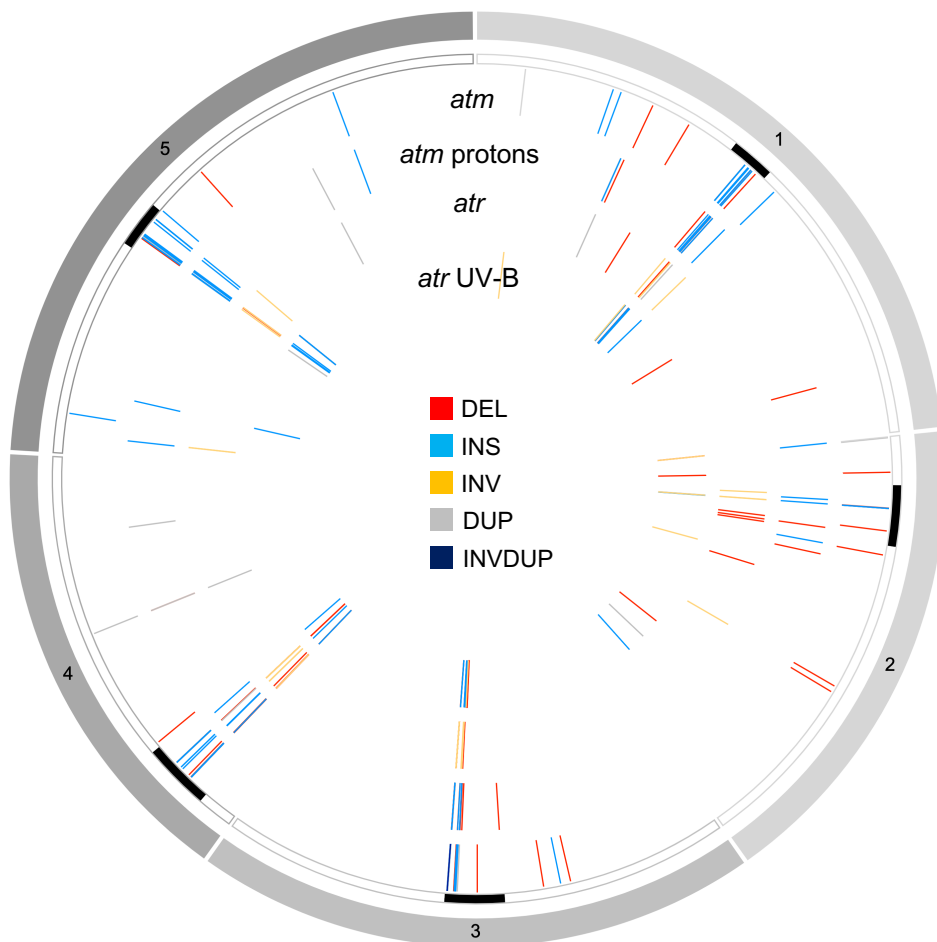

Figure S7

**a**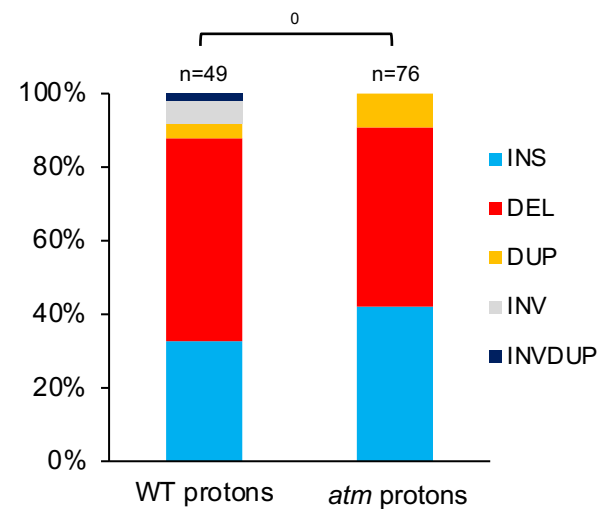**c**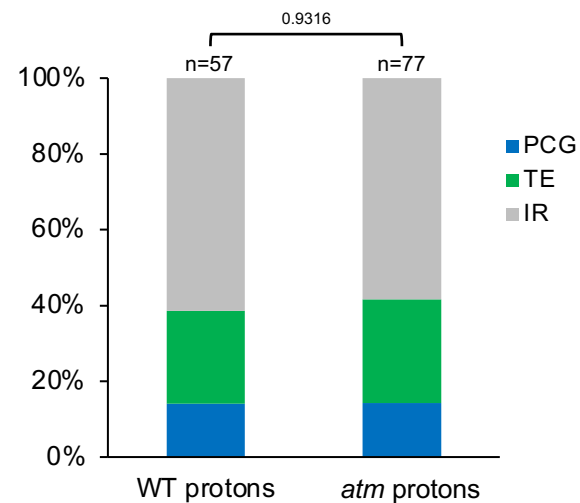**e**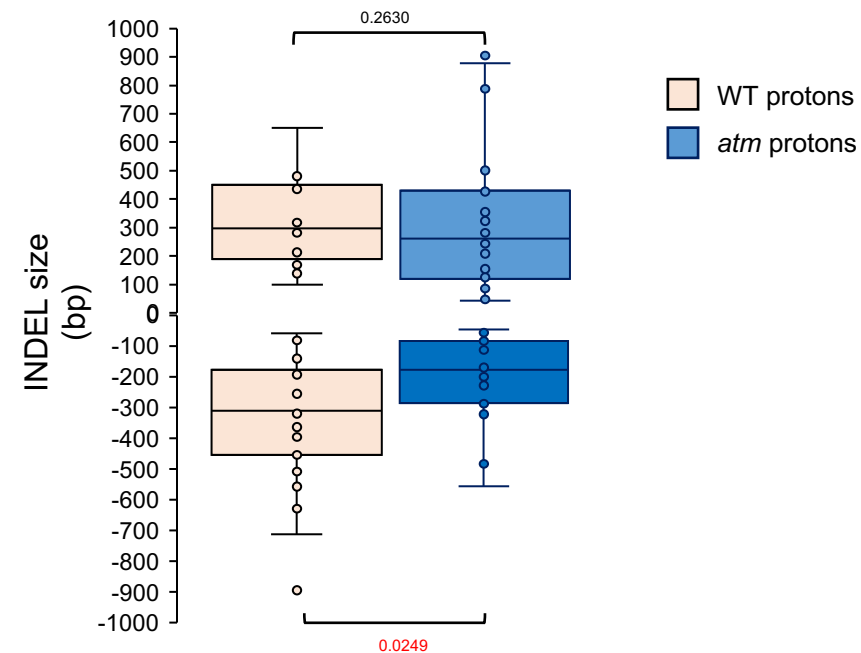**b**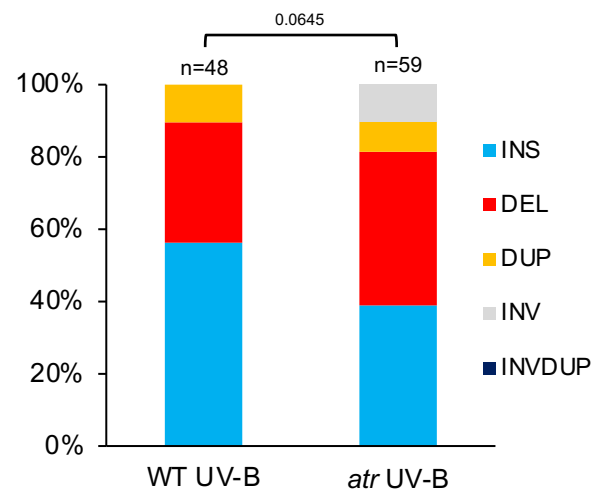**d**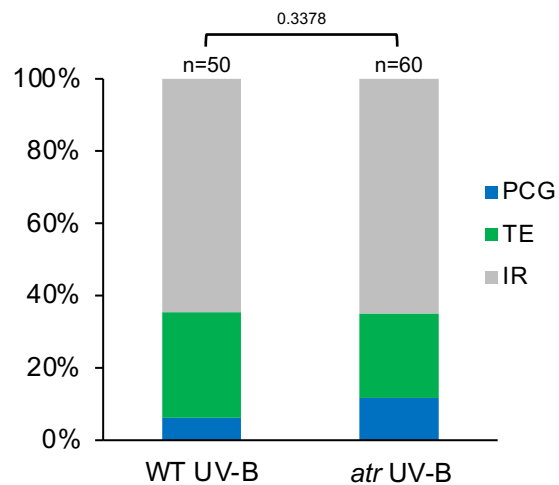**f**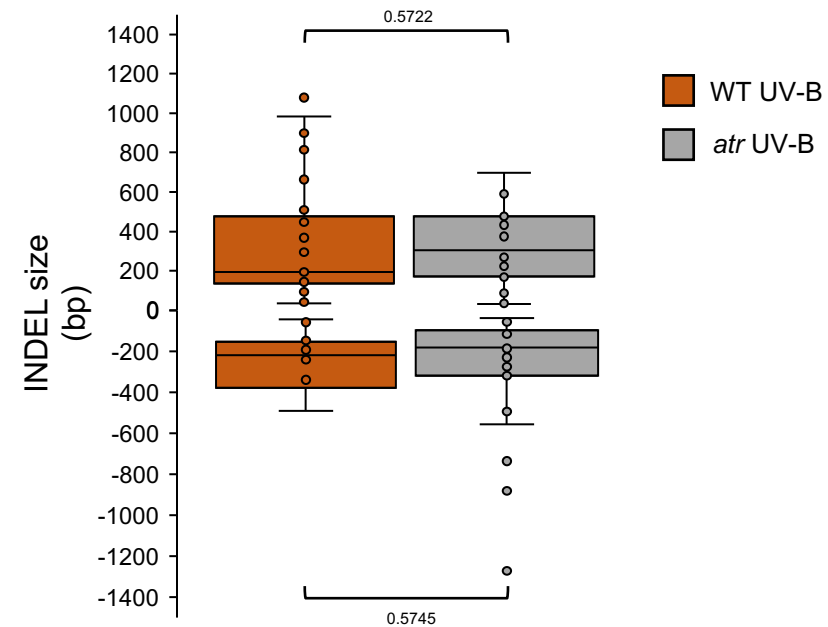

Figure S8

Supplement: Supplementary file 1 — Figure S1. TE superfamilies exhibiting SV in WT Arabidopsis plants. Figure S2. TE superfamilies exhibiting SV in radiation‐treated WT Arabidopsis plants. Figure S3. INDEL size in genetic elements of radiation‐treated WT Arabidopsis plants. Figure S4. Origins of insertions in WT‐irradiated Arabidopsis plants. Figure S5. Origins of insertions in radiation‐treated WT Arabidopsis plants. Figure S6. Genomic SV overlapping with HOT regions. Figure S7. Genomic locations of structural variations. Figure S8. Comparisons of the radiation‐induced genomic structural variations between WT, atr and atm Arabidopsis plants. [file TPJ-121-0-s001.zip › All Supp fig combined.pdf]
