# Supplementary material for: Characterization of radiations‐induced genomic structural variations in Arabidopsis thaliana
Source: Plant J. 2024 Dec 1;121(1):e17180. doi: 10.1111/tpj.17180 (PMC11712536; doi:10.1111/tpj.17180)
Supplement: Supplementary file 3 — Table S2. Sequencing statistics. [file TPJ-121-0-s002.docx]

**Supplemental Table 2:** Sequencing statistics

| **Sample** | **Genotype** | **Treatment** | **Mean read**  **length**  **(bp)** | **Mean read**  **quality** | **Median read**  **Length**  **(bp)** | **Median read**  **quality** | **Read length N50 kb** | **Total Gb** | **Number of reads**  **(x 1,000)** | **Mapped fraction** | **Average coverage** |
| --- | --- | --- | --- | --- | --- | --- | --- | --- | --- | --- | --- |
| **WT rep1** | WT | no | 4,754.8 | 9.7 | 1,007 | 10.1 | 25.62 | 5.00 | 1,152.94 | 0.63 | 22.89 |
| **WT rep2** | WT | no | 4,073.9 | 13.6 | 884 | 14.2 | 19.93 | 10.53 | 2,900.00 | 0.82 | 60.56 |
| **WT rep3** | WT | no | 5,555.6 | 13.1 | 856 | 13.7 | 25.14 | 5.04 | 908.22 | 0.81 | 28.63 |
| **WT UV-B** | WT | UV-B | 3,160.7 | 9.6 | 1,028 | 10 | ﻿11.18 | 10.38 | ﻿3,290.00 | 0.88 | 55.62 |
| **WT UV-C** | WT | UV-C | 5,524.2 | 9.8 | 1,485 | 10.1 | ﻿21.24 | 10.47 | ﻿1,895.53 | 0.86 | 60.80 |
| **WT Protons** | WT | Protons | 5,462.3 | 9.9 | 1,151 | 10.3 | ﻿21.90 | ﻿3.87 | ﻿4,098.09 | 0.90 | 51.88 |
| ***atm*** | *atm* | no | 6,432.5 | 9.7 | 1,052 | 10.1 | 26.87 | 2.10 | 2,485.28 | 0.50 | 12.90 |
| ***atr*** | *atr* | no | 3,724.7 | 9.1 | 714 | 9.5 | 23.23 | ﻿13.92 | ﻿3,883.81 | 0.41 | 76.75 |
| ***atm atr*** | *atm atr* | no | 8,754.7 | 12.6 | 5,965 | 12.4 | 14.00 | 11.88 | 861.426 | 0.93 | 41.45 |
| ***atm* Protons** | *atm* | Protons | 5,804.1 | 9.2 | 1,079 | 9.6 | 30.58 | ﻿10.95 | ﻿2,061.43 | 0.63 | 66.50 |
| ***atr* UV-B** | *atr* | UV-B | 5,702.7 | 9.1 | 905 | 9.5 | ﻿29.48 | ﻿10.97 | ﻿2,139.53 | 0.58 | 66.38 |
